# Supplementary material for: Frustrated charge density wave and quasi-long-range bond-orientational order in the magnetic kagome FeGe
Source: Nat Commun. 2025 May 1;16:4091. doi: 10.1038/s41467-025-58725-2 (PMC12045966; doi:10.1038/s41467-025-58725-2)
Supplement: Supplementary file 2 — Supplementary Information [file 41467_2025_58725_MOESM2_ESM.pdf]

Supplementary Information for ‘Frustrated charge density wave and quasi-long-range bond-orientational order in the magnetic kagome FeGe’

D. Subires,[1,2] A. Kar,[1] A. Korshunov,[1] C. A. Fuller,[3] Y. Jiang,[1] H. Hu,[1] D. Călugăru,[4] C. McMonagle,[3] C. Yi,[5] S. Roychowdhury,[5,6] W. Schnelle,[7] C. Shekhar,[7] J. Strempfer,[8] A. Jana,[9,10] I. Vobornik,[9] J. Dai,[11] M. Tallarida,[11] D. Chernyshov,[3] A. Bosak,[12] C. Felser,[7,*] B. Andrei Bernevig,[1,4,13] and S. Blanco-Canosa[1,13,†]

[1]*Donostia International Physics Center (DIPC), Paseo Manuel de Lardizábal, E-20018, San Sebastián, Spain*
[2]*Departamento de Física Aplicada I, Universidad del País Vasco UPV/EHU, E-20018 San Sebastián, Spain*
[3]*Swiss-Norwegian BeamLines at European Synchrotron Radiation Facility, BP 220, F-38043 Grenoble Cedex, France*
[4]*Department of Physics, Princeton University, Princeton, New Jersey 08544, USA*
[5]*Max Planck Institute for Chemical Physics of Solids, 01187 Dresden, Germany*
[6]*Department of Chemistry, Indian Institute of Science Education and Research Bhopal, Bhopal-462 066, India*
[7]*Max Planck Institute for Chemical Physics of Solids, D-01187 Dresden, Germany*
[8]*Advanced Photon Source, Argonne National Laboratory, Lemont, IL 60439, USA*
[9]*CNR-Istituto Officina dei Materiali (CNR-IOM), Strada Statale 14, km 163.5, 34149 Trieste, Italy*
[10]*International Center for Theoretical Physics (ICTP), Strada Costiera 11, 34151 Trieste, Italy*
[11]*ALBA Synchrotron Light Source, E-08290 Barcelona, Spain*
[12]*European Synchrotron Radiation Facility (ESRF), BP 220, F-38043 Grenoble Cedex, France*
[13]*IKERBASQUE, Basque Foundation for Science, E-48013 Bilbao, Spain*

CONTENTS

Supplementary Note 1. Sample growth and characterization 2

Supplementary Note 2. x-ray diffraction 2

Supplementary Note 3. Angle Resolved Photoemission: ARPES 4

Supplementary Note 4. First-principle calculation methods 6

Supplementary Note 5. Bulk band structure in non-CDW and CDW phases 6

Supplementary Note 6. Surface band structure in the CDW phase 6

Supplementary Note 7. Diffuse scattering, DS 13
- A. DS in as-grown FeGe 15
- B. Annealed FeGe 15
- C. $FeGe_{0.9}$ 19
- D. FeSn 22

Supplementary Note 8. Monte Carlo simulation of DS 23
- A. Ising model for Ge dimerization 23
- B. Monte Carlo simulations 23

Supplementary Note 9. Bond order (BO) correlation analysis 26

Supplementary References 29

* Claudia.Felser@cpfs.mpg.de

† sblanco@dipc.org

Supplementary Note 1. SAMPLE GROWTH AND CHARACTERIZATION

Single crystals of FeGe were grown by the chemical vapor transport method (see Methods) and characterized using energy dispersion x-ray (EDX) analysis, Laue diffraction, resistivity, and magnetization (Supplementary Fig. 1).

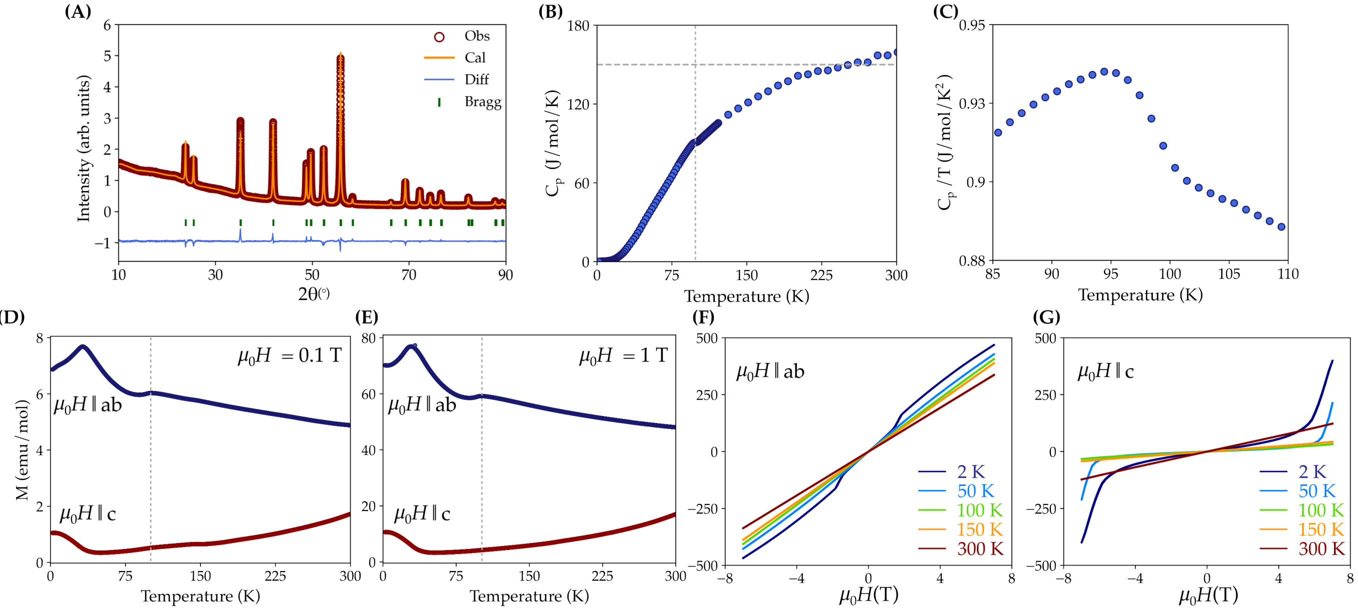

Supplementary Figure 1. Characterization of FeGe. (A) Powder x-ray diffraction refinement and its Le Bail refinement. (B) Temperature dependence of the heat capacity. (C) Heat capacity is divided by temperature, highlighting the CDW phase transition. (D-E) Temperature dependence of the magnetization for applied magnetic fields of 0.1 T and 1 T, respectively. (F-G) Magnetization versus magnetic field parallel (F) and perpendicular (G) to the Kagome plane.

Supplementary Fig. 1 (A) shows the Le Bail refinement of grounded FeGe single crystals. No impurity phases were observed, especially the presence of the cubic B20 phase. Both the specific heat (C_p), Supplementary Figs. 1 (B-C) and the magnetization, Supplementary Figs. 1 (D-E), identify the CDW transition at $\sim$ 105K. The transport properties are in good agreement with the reports in the literature [1, 2].

Supplementary Note 2. X-RAY DIFFRACTION

This section contains the single crystal structural refinement of FeGe at 80 K. Both *P6mm* (non-centrosymmetric), Supplementary Table I, and *P6/mmm* (centrosymmetric) Supplementary Table II could be equally indexed with similar *Goodness-of-Fit*. The x-ray refinements were carried out with the SHELXL2018/1 code (see Methods).

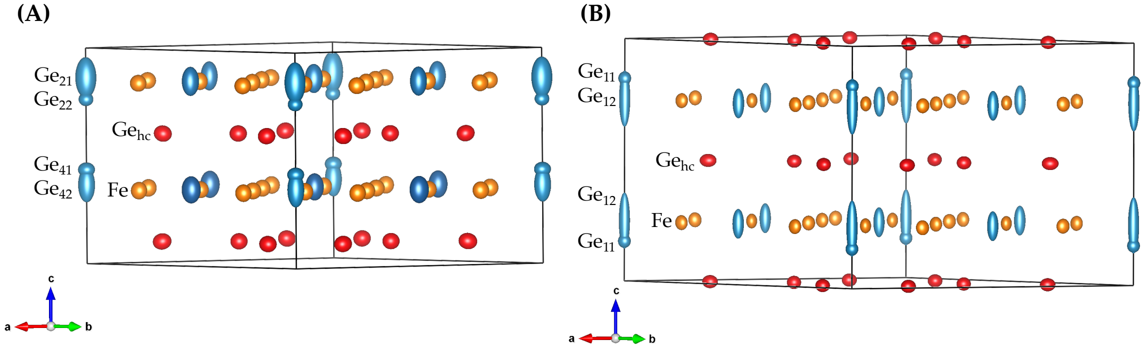

Supplementary Figure 2. (A) CDW unit cell of the space group 183 and (B) space group 191. The orange spheres represent the Fe atoms, red spheres are the Ge atoms within the honeycomb layer and the blue spheres (or ellipsoids) are the Ge atoms within the kagome plane.

Supplementary Fig. 2 displays the low-temperature CDW unit cell within the space group 183 (non-centrosymmetric *P6mm*) and 191 (centrosymmetric *P6/mmm*). The dimerized trigonal Ge in the space group 183 creates 4 in-equivalent trigonal Ge in the kagome plane (Ge_{21}, Ge_{22}, Ge_{41} and Ge_{42}), while the dimerization on the space group 191 splits the trigonal Ge into 2 Ge_{11} and 2 Ge_{12}.

Supplementary Table I. Crystal data and structure refinement for 80 K: *P6mm* symmetry, space group no= 183. Unit cell dimensions *a* = *b* = 9.97740(2) Å, *c* = 8.10070(10) Å, α=β= 90º, γ = 120º. Volume =698.3738(20) Å^3. R_1=0.056

		x	*y*	*z*	Occ.	U	Site	Sym.
Ge	Ge_1	0.33333	0.66667	0.60251	1.000	0.012	2b	3m.
Ge	Ge_{22}	1.00000	1.00000	0.76210	0.309	0.009	1a	6mm
Ge	Ge_{21}	1.00000	1.00000	0.86285	0.691	0.036	1a	6mm
Ge	Ge_3	0.67051	0.83526	0.10257	1.000	0.013	6e	.m.
Ge	Ge_{41}	1.00000	1.00000	0.43604	0.378	0.011	1a	6mm
Ge	Ge_{42}	1.00000	1.00000	0.36518	0.622	0.025	1a	6mm
Ge	Ge_5	0.66412	0.83206	0.60263	1.000	0.013	6e	.m.
Ge	Ge_6	0.33333	0.66667	0.10276	1.000	0.012	2b	3m.
Ge	Ge_7	0.50000	1.00000	0.85698	1.000	0.020	3c	2mm
Ge	Ge_8	0.50000	1.00000	0.35434	1.000	0.020	3c	2mm
Fe	Fe_1	0.74954	1.00000	0.34468	1.000	0.012	6d	..m
Fe	Fe_2	0.50020	0.75010	0.84475	1.000	0.012	6e	.m.
Fe	Fe_3	0.49999	0.75000	0.34241	1.000	0.012	6e	.m.
Fe	Fe_4	0.74963	1.00000	0.84234	1.000	0.012	6d	..m

Supplementary Table II. Crystal data and structure refinement for 80 K: *P6/mmm* symmetry, space group no= 191. Unit cell dimensions *a* = *b*=9.97750(10) Å, *c* = 8.10030(10) Å, α=β= 90º, γ = 120º. Volume =698.353(16) Å^3, R_1=0.056

		x	*y*	*z*	Occ.	U	Site	Sym.
Fe	Fe_1	0.25053	0.00000	0.75162	1.000	0.004	12n	..m
Fe	Fe_2	0.24996	0.49991	0.74843	1.000	0.004	12o	.m.
Ge	Ge_{11}	0.00000	0.00000	0.83590	0.485	0.004	2e	6mm
Ge	Ge_{12}	0.00000	0.00000	0.75170	0.515	0.028	2e	6mm
Ge	Ge_2	0.50000	0.00000	0.74842	1.000	0.009	6i	2mm
Ge	Ge_3	0.16844	0.33687	0.00000	1.000	0.005	6l	mm2
Ge	Ge_4	0.83593	0.67186	0.50000	1.000	0.005	6m	mm2
Ge	Ge_5	0.33333	0.66667	0.00000	1.000	0.005	2c	-6m2
Ge	Ge_6	0.66667	0.33333	0.50000	1.000	0.005	2d	-6m2

Supplementary Note 3. ANGLE RESOLVED PHOTOEMISSION: ARPES

We observe the following key features in the experimental band structure from ARPES:

1. A clear Dirac crossing appears at K at about -0.65 eV.
2. A V-shaped band centered at Γ with the bottom at about -1.0 eV.
3. A very broad spectrum weight connects Γ and the Dirac crossing at K, which could be the weights from the Dirac bands and together with other bands.
4. A U-shaped band centered at Γ with the bottom at about -0.28 eV.

The first two features in ARPES, i.e., the Dirac crossing at K and the V-shaped band at Γ could be well-matched using the CDW bulk band structure in DFT, as shown in Supplementary Fig. 3. The broad spectrum weight connects Γ and at K is also seen in DFT, although the energy in DFT is slightly higher. However, the U-shape is not seen in the bulk bands. We argue that it can be matched using the surface bands in the honeycomb Ge termination, as shown in Supplementary Fig. 3.

The slight disagreement between our ARPES data and the DFT calculations could have its origin in the improper treatment of dimerized and undimerized phases in DFT and the multiorbital character of the Fe. These include: (i) In DFT, there exists another Dirac-like crossing at K at about -0.4 eV, which is not seen in the APRES. (ii) The broad weight at -1 eV near Γ in DFT. (iii) In the folded surface bands, there exist many other bands that could not have a good match with the APRES, probably due to the inaccurate description of surface instructions in DFT. We conclude that the main features in the experimental ARPES bands could be well-matched by DFT. However, since the system has a large number of bands near the Fermi level, it shows a heavily broadened spectrum where the discrepancy in the detailed features between theory and experiments is expected.

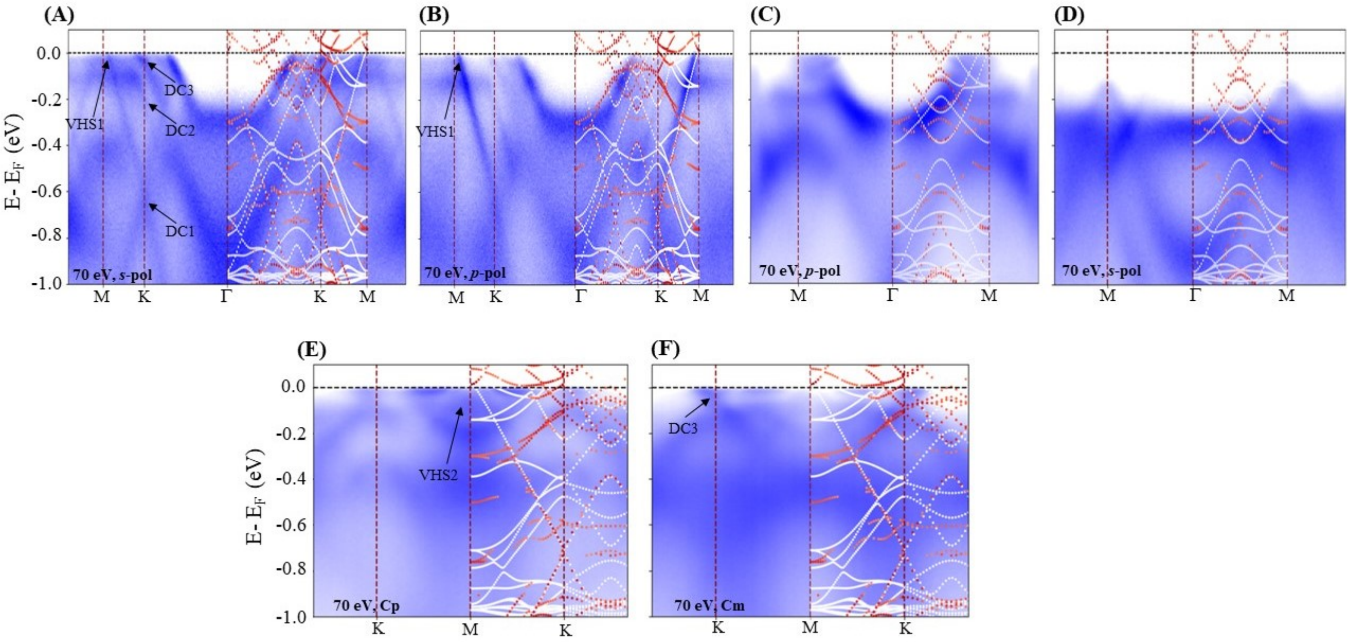

Supplementary Figure 3. A comparison between ARPES and DFT calculated energy dispersion spectra. Valance band electronic structure of FeGe obtained with 70 eV incident photon energy along (A)-(B) $\mathrm{M}-\mathrm{K}-\Gamma-\mathrm{K}-\mathrm{M}$ symmetry direction with s-pol and p-polarized incident light, respectively. (C)-(D) along $\mathrm{M}-\Gamma-\mathrm{M}$ symmetry direction with p-pol and s-polarized light, respectively. (E)-(F) Along $\mathrm{K}-\mathrm{M}-\mathrm{K}$ symmetry direction with circular positive (C_p) and circular negative (C_m) incident light, respectively. ('DC' and 'VHS' represent the Dirac cone, and Van Hove singularity, respectively). On top of the VB spectra, DFT calculated bulk folded band structure in the CDW phase (white) and Ge terminated surface band structure (red) are overlapped.

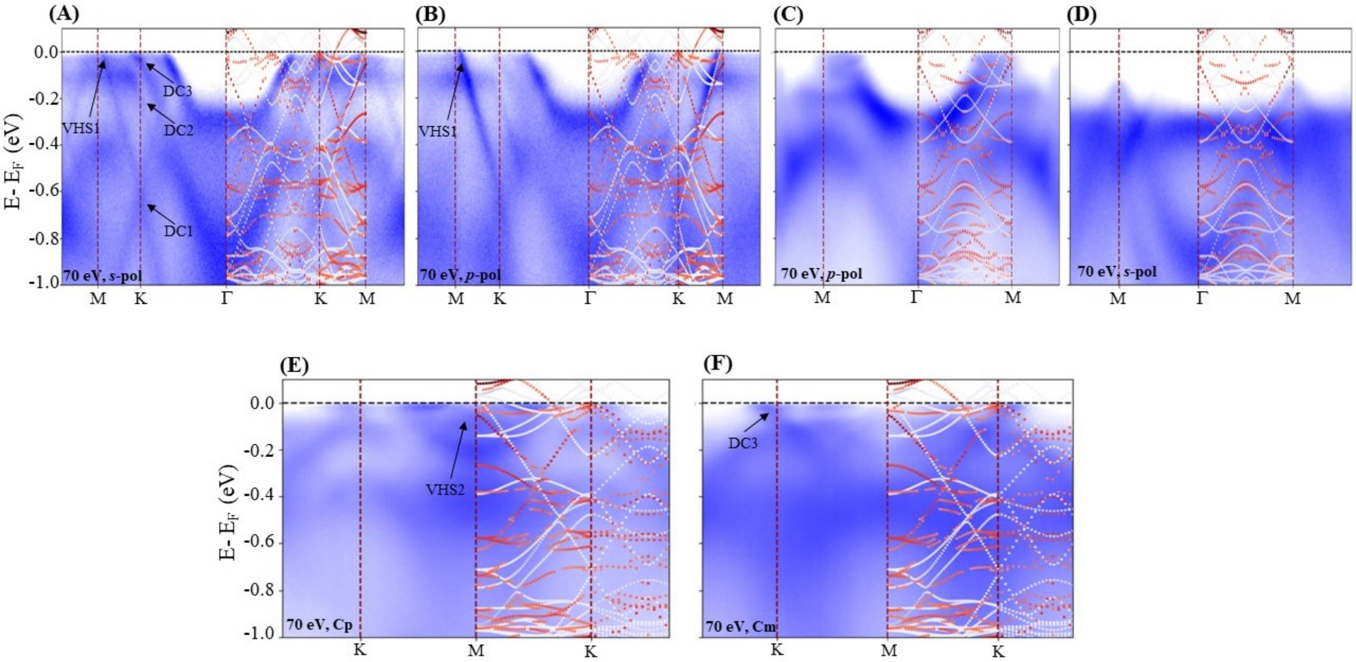

Supplementary Figure 4. A comparison between ARPES and DFT calculated spectra. The valance band specters of FeGe were obtained with 70 eV incident photon energy along (A)-(B) $\mathrm{M}-\mathrm{K}-\Gamma-\mathrm{K}-\mathrm{M}$ symmetry direction with s-pol and p-polarized incident light, respectively. (C)-(D) along $\mathrm{M}-\Gamma-\mathrm{M}$ symmetry direction with p-pol and s-polarized light, respectively. (E)-(F) Along $\mathrm{K}-\mathrm{M}-\mathrm{K}$ symmetry direction with circular positive (C_p) and circular negative (C_m) incident light, respectively. ('DC' and 'VHS' represent the Dirac cone and Van Hove singularity, respectively). Here, the 'white' bands are folded bulk bands of FeGe calculated in the CDW phase where whereas the 'red' ones are the unfolded surface bands obtained with Kagome termination.

Supplementary Note 4. FIRST-PRINCIPLE CALCULATION METHODS

The first-principle calculations in this work use the Vienna ab initio Simulation Package (VASP)[3–7] with generalized gradient approximation of Perdew-Burke-Ernzerhof (PBE) exchange-correlation potential[8]. A $8 \times 8 \times 8$ $(5 \times 5 \times 5)$ k-mesh for non-CDW (CDW) phase and an energy cutoff of 500 eV are used. The maximally localized Wannier functions are obtained using WANNIER90[9–12]. A local coordinate system at the kagome site is adopted to decompose d orbitals when constructing MLWFs, the same as the one used in Ref.[13]. The Wannier tight-binding models are symmetrized using *Wannhr_symm* in *WannierTools*[14]. The unfolding of CDW bands is performed using *VaspBandUnfolding* package[15, 16]. The Fermi surface is computed using *WannierTools*[14] and visualized using *Fermisurfer*[17].

Supplementary Note 5. BULK BAND STRUCTURE IN NON-CDW AND CDW PHASES

In this section, we discuss the bulk band structure in both non-CDW and CDW phases.

In Supplementary Fig. 5(a), the crystal structure of FeGe in the non-CDW phase is shown together with two surface terminations, i.e., kagome and honeycomb surfaces. In the $2 \times 2 \times 1$ CDW phase, the main atomic displacements come from the dimerization of the triangular Ge. Experimentally, the dimerized Ge atoms have two possible positions as shown in the main text. In DFT, we fix the Ge atoms at the larger dimerized positions.

In Supplementary Figs. 5(b) and (c), we show the comparison between the non-CDW and (unfolded) CDW bands in the PM and AFM phases. In Supplementary Fig. 8 and Supplementary Fig. 9, the orbital weights in the non-CDW AFM phase are shown, while in Supplementary Fig. 10, the orbital weights in the CDW phase are shown. We observe the following features in the CDW bulk bands:

- A Dirac crossing at K centered at about -0.7 eV mainly comes from d_{xy} orbitals, together with some $d_{x^2-y^2}$ weights. It exists in both CDW and non-CDW bands and has little changes, as shown in Supplementary Figs. 9(a) and (b) and Supplementary Fig. 10(b).

- A V-shaped band centered at Γ from about -0.5 to -1.0 eV mainly comes from the triangular Ge p_z orbital, as shown in Fig. 10(d). This band accounts for the main reconstruction by CDW. In the non-CDW phase, it is located at high energy with the bottom at about -0.5 eV, as shown in Supplementary Fig. 9(f).

- A quasi-flat bands at about -1 eV near Γ mainly comes from d_{z^2} orbital, as shown in Supplementary Fig. 9(c) and Supplementary Fig. 10(a).

In Supplementary Fig. 11, we also superimpose the CDW bulk bands with non-CDW bands for better comparison.

In Supplementary Fig. 6, we show the Fermi surface (FS) of FeGe in the non-CDW AFM phase. It can be seen that there is quasi-2D FS with a weak k_z-dispersion in Supplementary Fig. 6(c), which is mainly contributed by the $d_{x^2-y^2}, d_{xy}$ orbitals of Fe (see Supplementary Fig. 8). In Supplementary Fig. 7, we show the 2D slices of the FS on difference k_z planes. On the $k_z = 0$ plane, the smallest circular FS around Γ is mainly given by the p_z orbitals of the triangular Ge (see Supplementary Fig. 8(f)). In the CDW phase, this band moves down and is far from E_f (see Supplementary Fig. 10(d)). However, there are some other bands close to E_f near Γ in the CDW phase, which could contribute to the FSs (see Supplementary Fig. 11).

In Supplementary Fig. 12, we show the nesting function (Im-χ) and total susceptibility (Re-χ) of FeGe in the AFM phase (non-CDW). In the nesting function, the dominant peak appears at the K point. In the total susceptibility, however, a broad peak appears along the boundary of the first BZ, with the highest point near K. Thus we conclude that the FS nesting cannot directly account for the CDW at M point.

Supplementary Note 6. SURFACE BAND STRUCTURE IN THE CDW PHASE

In this section, we discuss the surface bands in the CDW phase. In Supplementary Fig. 13, the folded surface bands for both honeycomb and kagome terminations are given, together with orbital weights. In Supplementary Fig. 14, we also give the unfolded surface bands. Here the folded bands denote the bands in the CDW BZ, while unfolded bands denote those in the non-CDW BZ.

We observe the major difference between folded and unfolded surface bands is a U-shaped band centered at Γ at about -0.3 eV at the honeycomb surface. This U-shaped band only appears in the folded bands, which mainly comes from (d_{xz}, d_{yz}) of Fe. Since this band is not seen in the unfolded bands near Γ, it is folded from the M point due to the 2×2 CDW order. In the CDW bulk bands, there exist bands with a similar shape from (d_{xz}, d_{yz}) near $L = (\frac{1}{2}, 0, \frac{1}{2})$, as shown in Supplementary Fig. 10(g). Thus this surface U-shaped band can be seen as k_z-projected bulk bands with surface reconstructions.

This U-shaped band matches well with APRES results. We conjecture that there are strong disorder effects near the surface that break the translational symmetry. Thus the observed bands in ARPES can be explained by the folded surface bands.

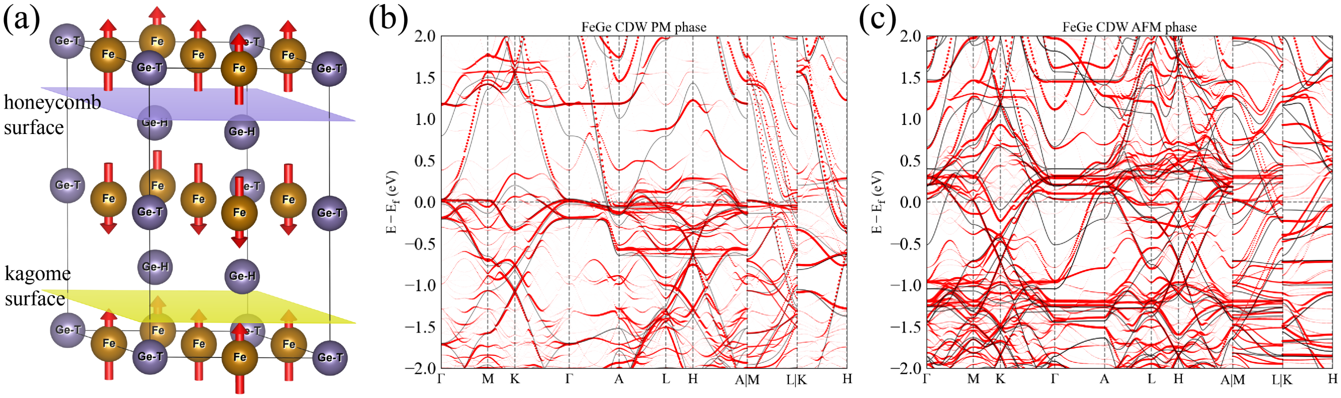

Supplementary Figure 5. (a) The crystal structure of FeGe in the non-CDW phase, where Fe atoms form Kagome lattices, while Ge atoms form triangular and honeycomb lattices (denoted as Ge-T and Ge-H, respectively). Two surface terminations, i.e., Kagome and honeycomb surfaces are also marked, where the atoms below the plane define the surface. (b) The band structures in non-CDW (black lines) and CDW (red lines) phases, where paramagnetic (PM) order is assumed. In the CDW phase, the bands are unfolded to the non-CDW Brillouin zone (BZ). (c) Same as (b), but in the anti-ferromagnetic (AFM) phase. Spin-orbital coupling (SOC) is neglected for simplicity as SOC is weak in FeGe.

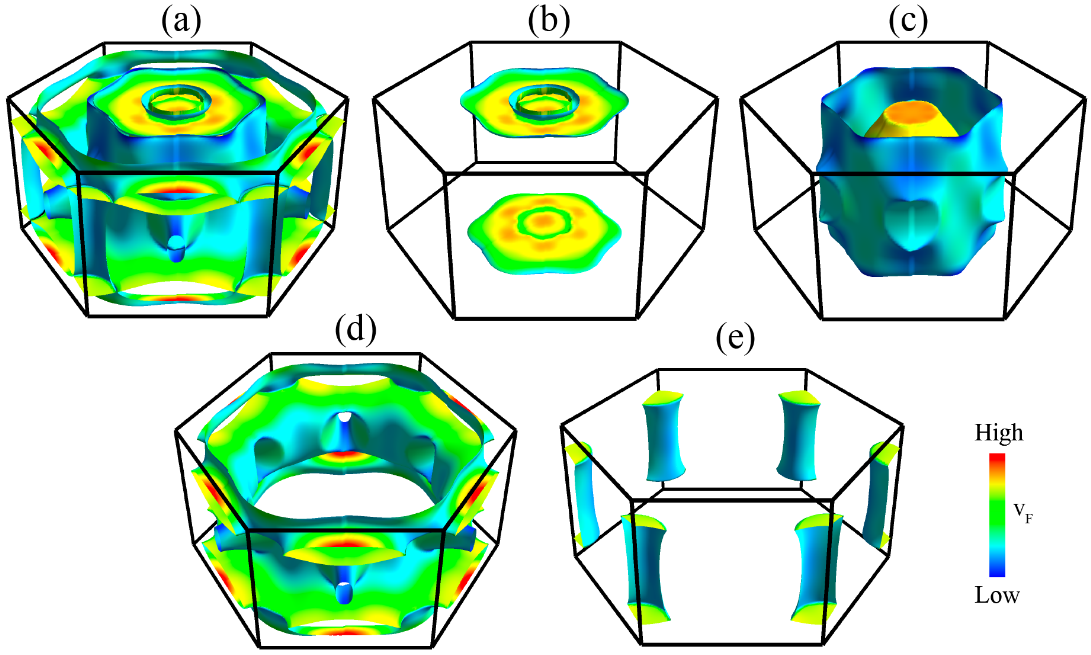

Supplementary Figure 6. The Fermi surface (FS) of FeGe in the non-CDW AFM phase, where (a) is the full FS, and (b)-(e) are four parts of FSs contributed by different bands. The color on the FS denotes the Fermi velocity. A quasi-2D FS is shown in (c) with a weak k_z-dispersion.

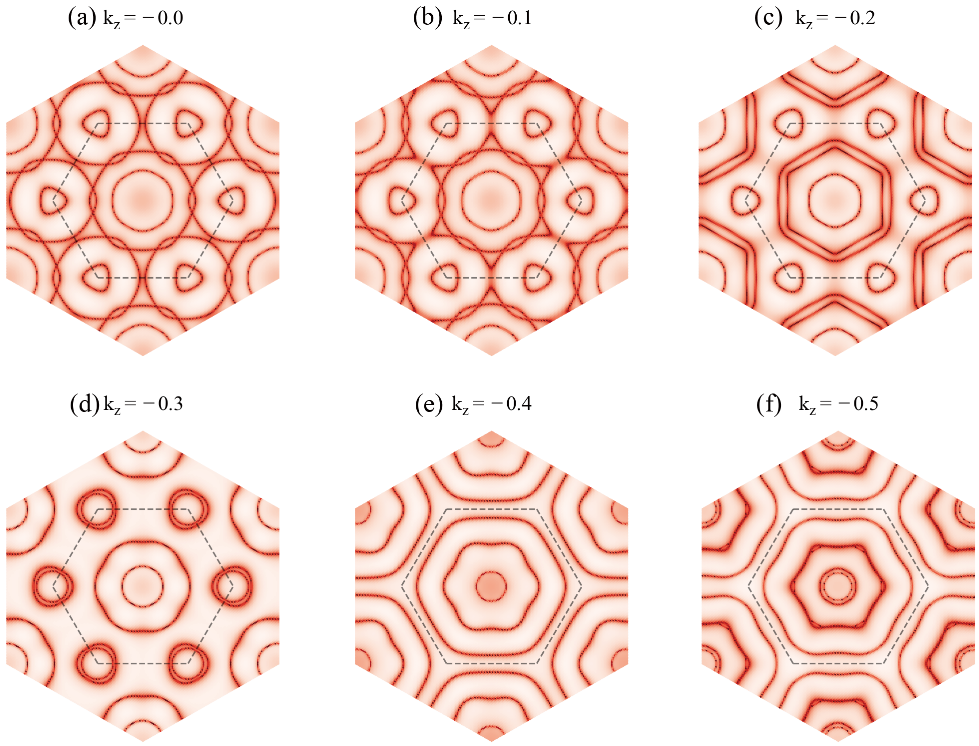

Supplementary Figure 7. The 2D Fermi surface (FS) of FeGe in the non-CDW AFM phase on different k_z planes.

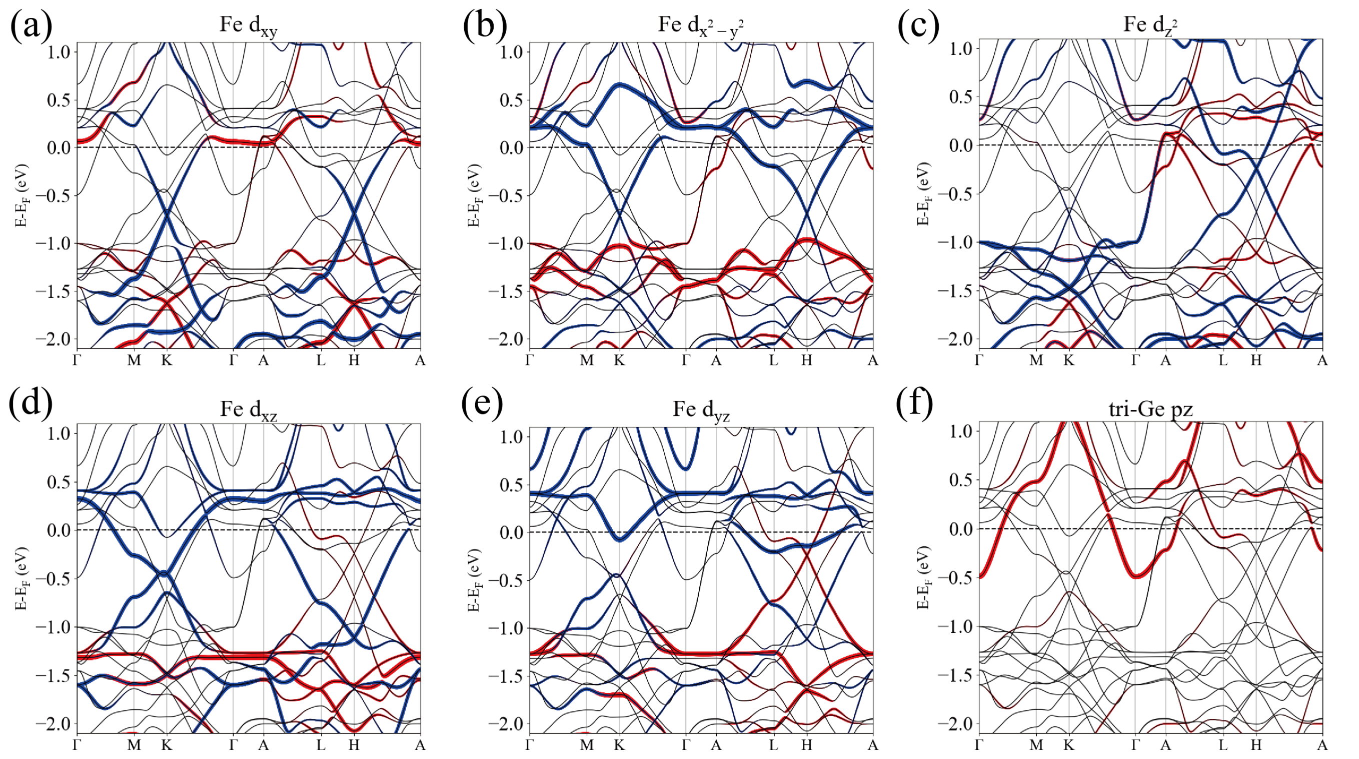

Supplementary Figure 8. The orbital projections in the non-CDW AFM phase, for Fe (a) d_{xy}, (b) $d_{x^2-y^2}$, (c) d_{z^2}, (d) d_{xz}, (e) d_{yz}, and triangular Ge p_z orbitals, respectively. Blue and red lines denote two spin-up and down bands from Fe one Kagome layer.

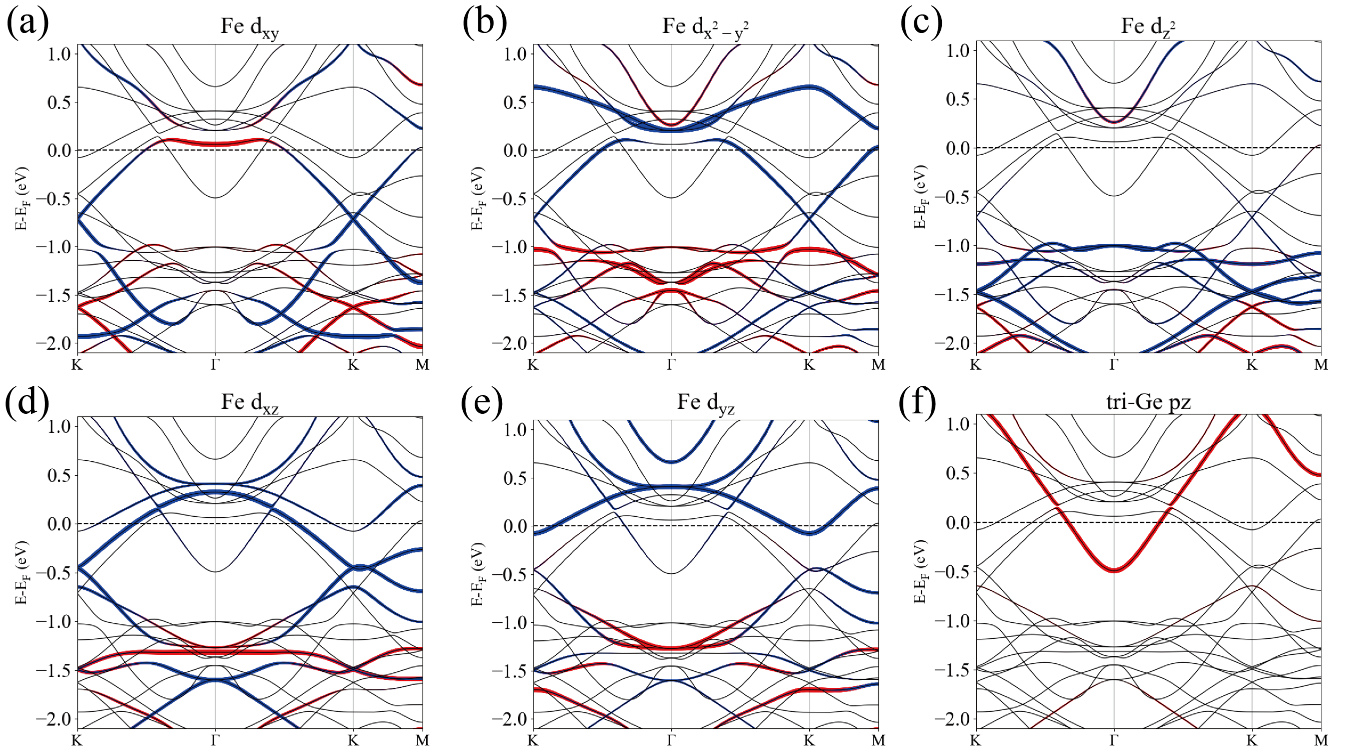

Supplementary Figure 9. The orbital projections in the non-CDW AFM phase. It is the same as Supplementary Fig. 8 but on a different path in the BZ, to give a direct comparison with ARPES results.

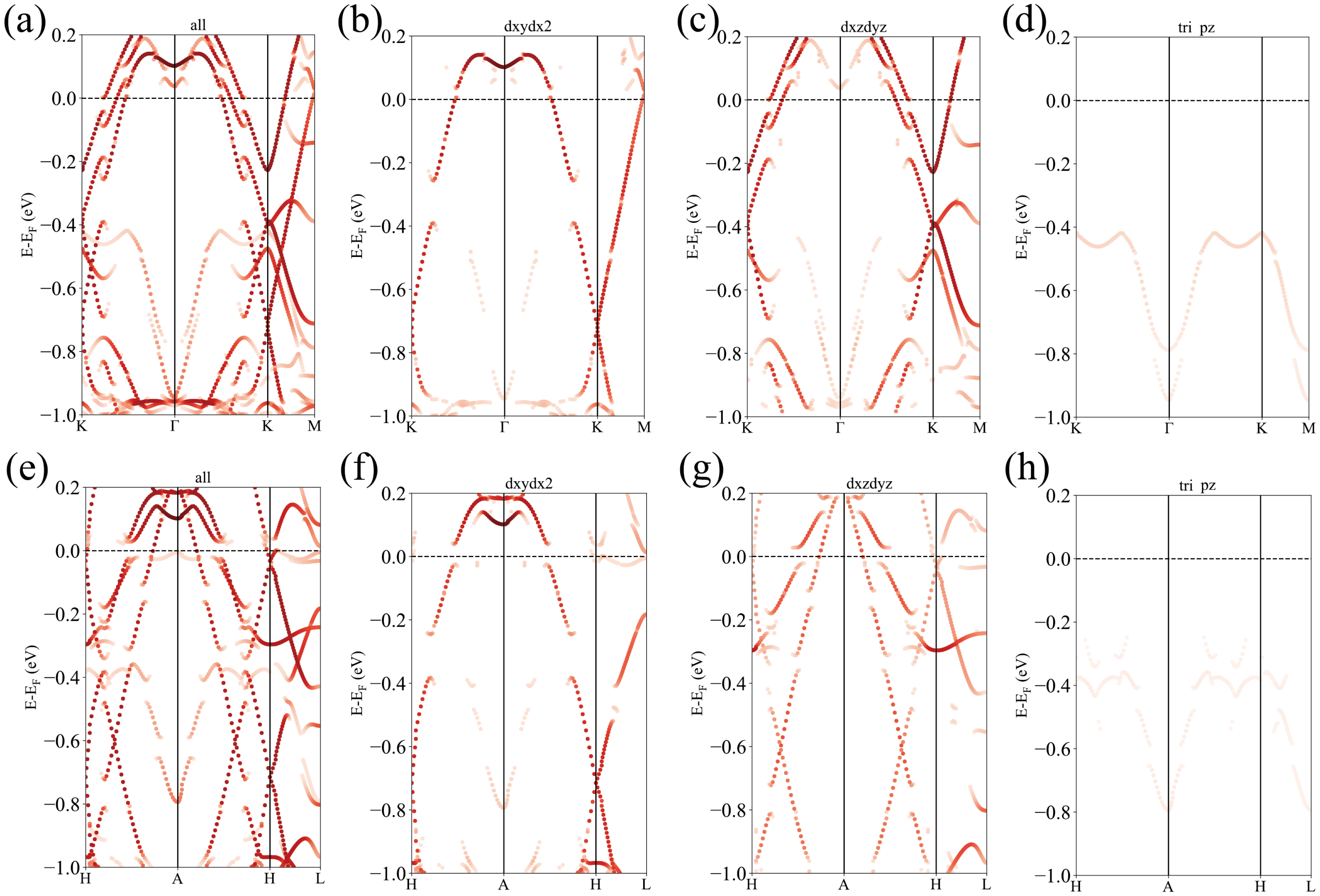

Supplementary Figure 10. The unfolded bulk band structures and orbital projections in the CDW AFM phase. (a) The unfolded bulk bands. (b)-(d) The orbital weights for Fe $(d_{xy}, d_{x^2-y^2})$, (d_{xz}, d_{yz}), and triangular Ge p_z orbitals, respectively. (e)-(h) in the second row is the same as the first row but on $k_z = \pi$ plane.

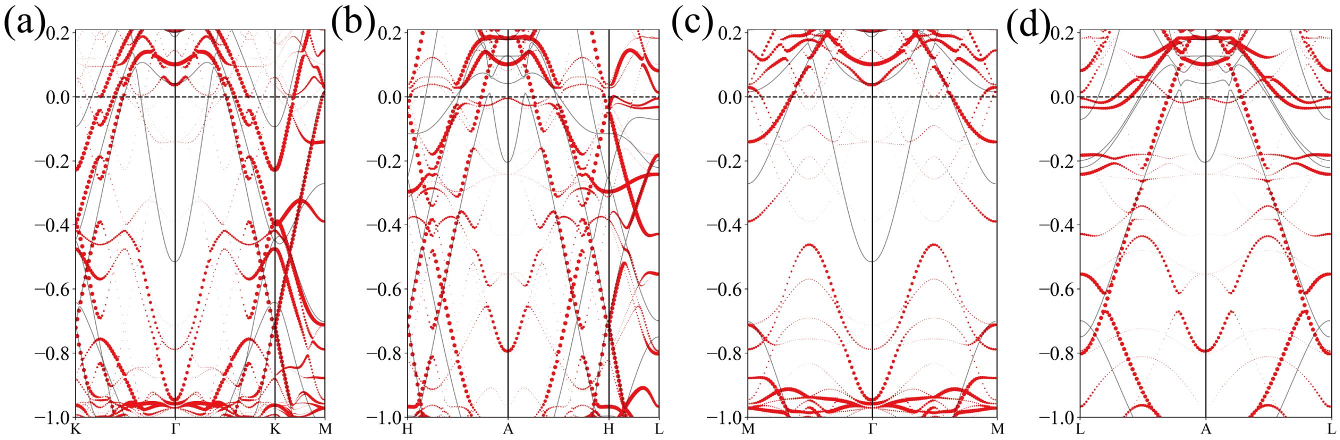

Supplementary Figure 11. Comparison of non-CDW (black) and unfolded CDW (red) bulk bands in the AFM phase, along four different paths in the BZ. The main difference between CDW and non-CDW bands is the V-shaped bands at Γ, which is mainly from triangular Ge p_z orbitals (see Fig. 9 and Fig. 10 for orbital weights). This band moves down in the CDW phase.

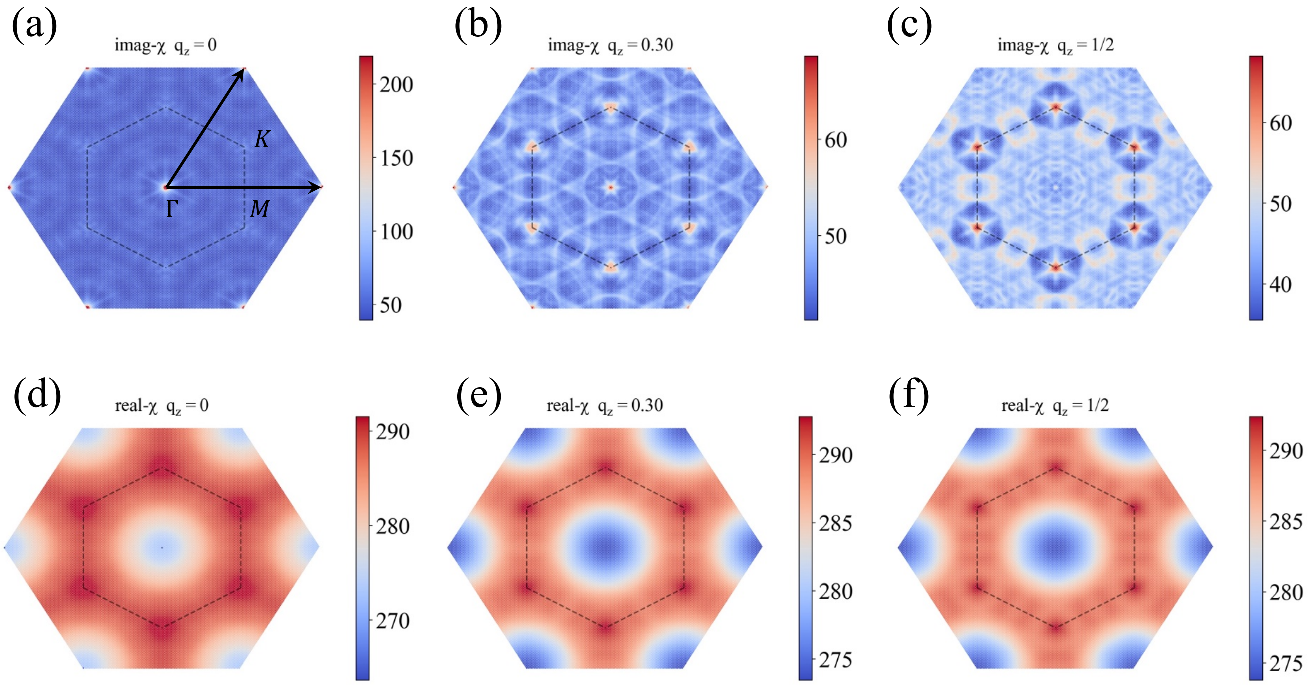

Supplementary Figure 12. The nesting function (Im-χ) and total susceptibility (Re-χ) of FeGe in the AFM phase (non-CDW). In the nesting function, the dominant peak appears at the K point. In the total susceptibility, however, a broad peak appears along the boundary of the first BZ, with the highest point near K.

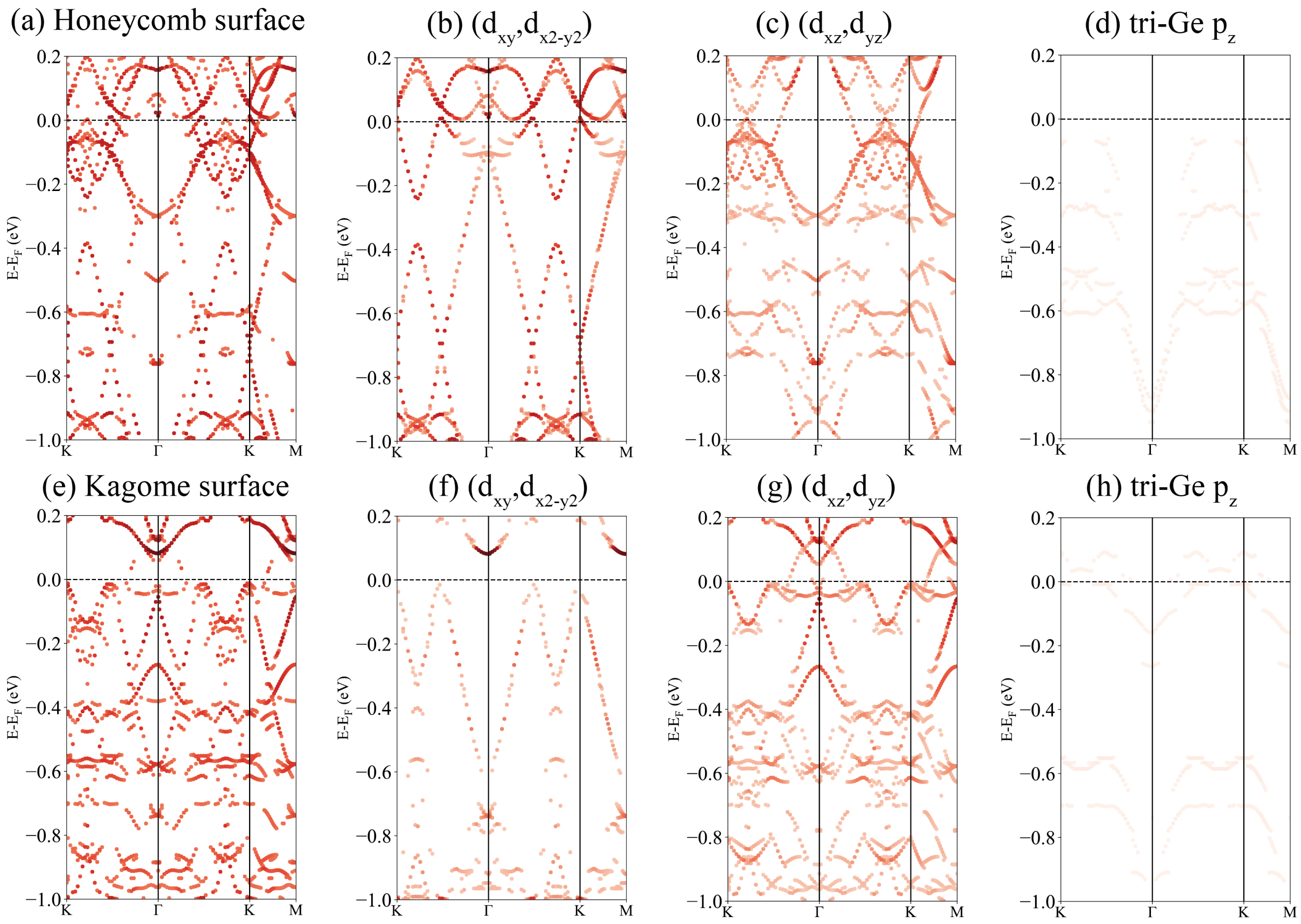

Supplementary Figure 13. The folded surface bands in the CDW phase. (a) The honeycomb surface bands. (b)-(d) The orbital weights in the honeycomb surface bands, for Fe $(d_{xy}, d_{d^2-y^2})$, (d_{xz}, d_{yz}), and triangular Ge p_z orbitals, respectively. A U-shaped band centered at Γ at about -0.3 eV is observed at the honeycomb surface which mainly comes from (d_{xz}, d_{yz}) of Fe. This band agrees with ARPES results. (e)-(h): same as (a)-(d) but for the Kagome surface. The definition of the two surface termination is given in Supplementary Fig. 5.

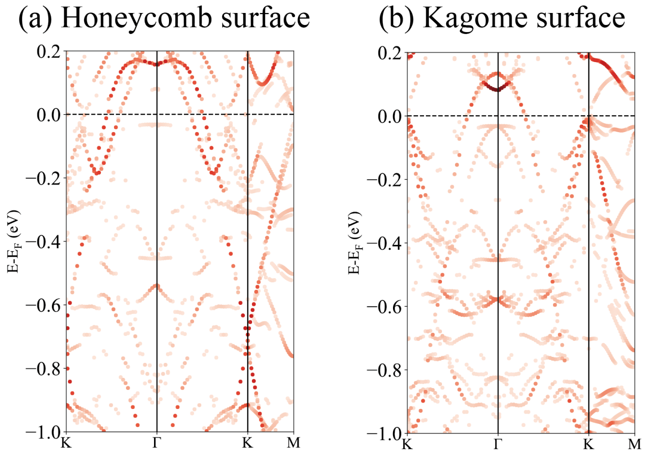

Supplementary Figure 14. The unfolded surface bands in the CDW phase, for both honeycomb surface (a) and Kagome surface (b).

Supplementary Note 7. DIFFUSE SCATTERING, DS

Single crystal's diffuse scattering contains information about local (short-range, pairs of sites) ordering in the sample and the deviations from the average (different types of disorder) at scales beyond the average unit cell. In this section, we present a comprehensive study of the different DS cuts of as-grown FeGe, annealed and Ge-deficient FeGe, and FeSn.

First of all, we will give a brief introduction about how to identify substitutional from displacement (i.e. phonon driven) disorder [18]. A system with the substitutional disorder can be characterized by a unit cell where a site is occupied by one of several atom types or a missing atom, thus the chemical environment in neighboring unit cells is not identical. Supplementary Figures 15 (A) and (B) show an example of a one-dimensional substitutional disordered chain with a random occupation per unit cell and two different types of atom types; i.e. Fe and Ge. If Fe and Ge are present in a fraction m_{Fe} and m_{Ge}, respectively, then:

$$m_{\mathrm{Fe}} + m_{\mathrm{Ge}} = 1, \tag{1}$$

In a system with positive correlated disorder, Fe or Ge coherently cluster together; e.g. neighboring atoms are of the same type, Supplementary Fig. 15 (A). If the disorder is negatively correlated if Fe and Ge alternate along the chain, e.g. Fe tends to have Ge as its neighbor, Supplementary Fig. 15 (B). Starting from a toy model, a binary disordered system is commonly characterized by the Warren-Cowley short-range order parameters $\alpha_{\vec{v}}$;

$$\alpha_{\vec{v}} = 1 - \frac{P_{\vec{v}}^{\mathrm{FeGe}}}{m_{\mathrm{Fe}} m_{\mathrm{Ge}}}, \tag{2}$$

-where $\mathrm{P}_{\vec{v}}^{\mathrm{FeGe}}$ describes the probability of finding a Ge atom from a Fe atom at a vector $\vec{v}$. The positive and negative correlations in a certain direction are, thus, parametrized by Warren-Cowley short-range order values.

$$\alpha_{\vec{v}} = \begin{cases} > 0 & \text{positive correlation,} \\ = 0 & \text{no correlation,} \\ < 0 & \text{negative correlation.} \end{cases} \tag{3}$$

The Warren-Cowley parameters are equivalent to the c_i coefficients obtained by DFT in the main text.

The diffraction patterns from a positive, negative, and uncorrelated one-dimensional chain are displayed in Supplementary Fig. 15 (C). The main observation is a decrease in intensity as a function of the momentum transfer. Moreover, positive correlation results in a large diffraction intensity at integer values of h, while a negative $\alpha_{\vec{v}}$ localizes the intensity at half-integer h (in between Bragg peaks).

In a system with pure displacement disorder, the atoms are displaced from their average position within the unit cell. This is the case for thermal disorder or the condensation of a particular phonon mode associated with a CDW phase transition. Therefore, it is strongly dependent on the phonon eigenvectors, the polarization, and the electron-phonon interaction. The displacement vector of an atom in a unit cell t on-site i from its average position is given by $\vec{\delta}_{t,i}$ and follows a Gaussian probability with a covariance matrix $\underline{u}$:

$$p(\vec{\delta}_{t,i}) = \frac{1}{\sqrt{(2\pi)^3 \det(\underline{u})}} \exp\left(-\frac{1}{2}(\vec{\delta}_{t,i})^T \underline{u}^{-1} (\vec{\delta}_{t,i})\right), \tag{4}$$

The displacement disorder is random if the displacement of an atom is independent of the displacement of its neighboring sites, and positively correlated if the displacement of neighboring atoms is preferably along the same direction or opposing directions (negative correlation). The diffuse diffraction pattern of a positive, a negative, and an uncorrelated system driven by displacement disorder is shown in Supplementary Fig. 16.

The signature of diffuse scattering caused by displacement disorder is a decrease of the maximum intensity at $h \to 0$, where the substitutional disorder develops its maximum intensity. Therefore, the momentum dependence of the diffuse scattering allows us to directly distinguish between displacement and substitutional disorder. This is the case observed in FeGe, where the DS follows the typical trend characteristic of substitutional disorder instead of phonon-driven.

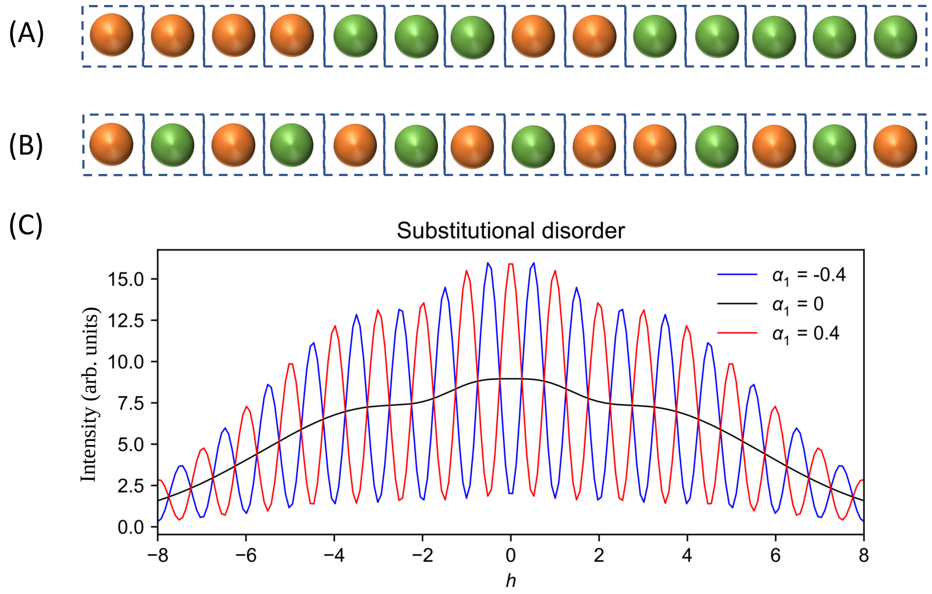

Supplementary Figure 15. (A) 1D-chain of Fe and Ge atoms with substitutional disorder, where (A) the same type of atoms (Fe or Ge) cluster together and (B) Fe and Ge type of atoms alternate in the chain. (C) Diffuse scattering pattern for substitutional disordered pattern. Note how the DS decreases with *h*.

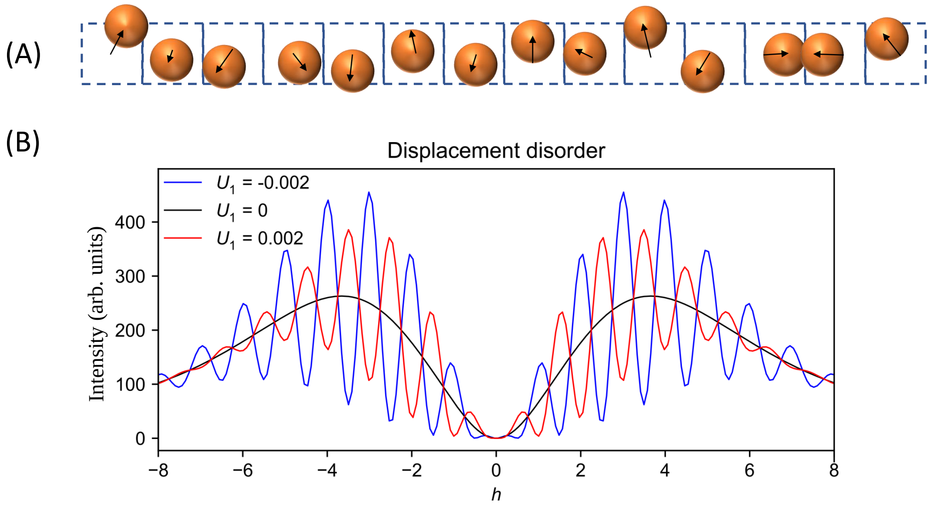

Supplementary Figure 16. (A) Diffuse scattering forms a one-dimensional Ge crystal with displacement disorder. In (A), the arrows stand for the atomic displacements. (B) Calculated DS for correlated (U positive, atoms move in-phase) and uncorrelated (U negative, atoms move out-of-phase). The DS is negligible at $h \rightarrow =0$, hence differentiating from the substitutional disorder case. Note that positive correlation gives maxima of DS at the Bragg positions, while negative correlation gives DS at half-integer h.

A. DS in as-grown FeGe

In Supplementary Figs. 17 (A-C), we show the (h k) complementary DS cuts of the main text for L=3, 1.5 and 2.5 planes of as-grown FeGe. Signatures of DS driven by substitutional disorder, namely hexagonal diffuse rings, are also visible in the (h k 3) plane. Similarly to the (h k 1.5), the (h k 2.5) plane shows a complex diffuse pattern, presumably as a result of the small in-plane atomic displacements, not considered in the MC simulations.

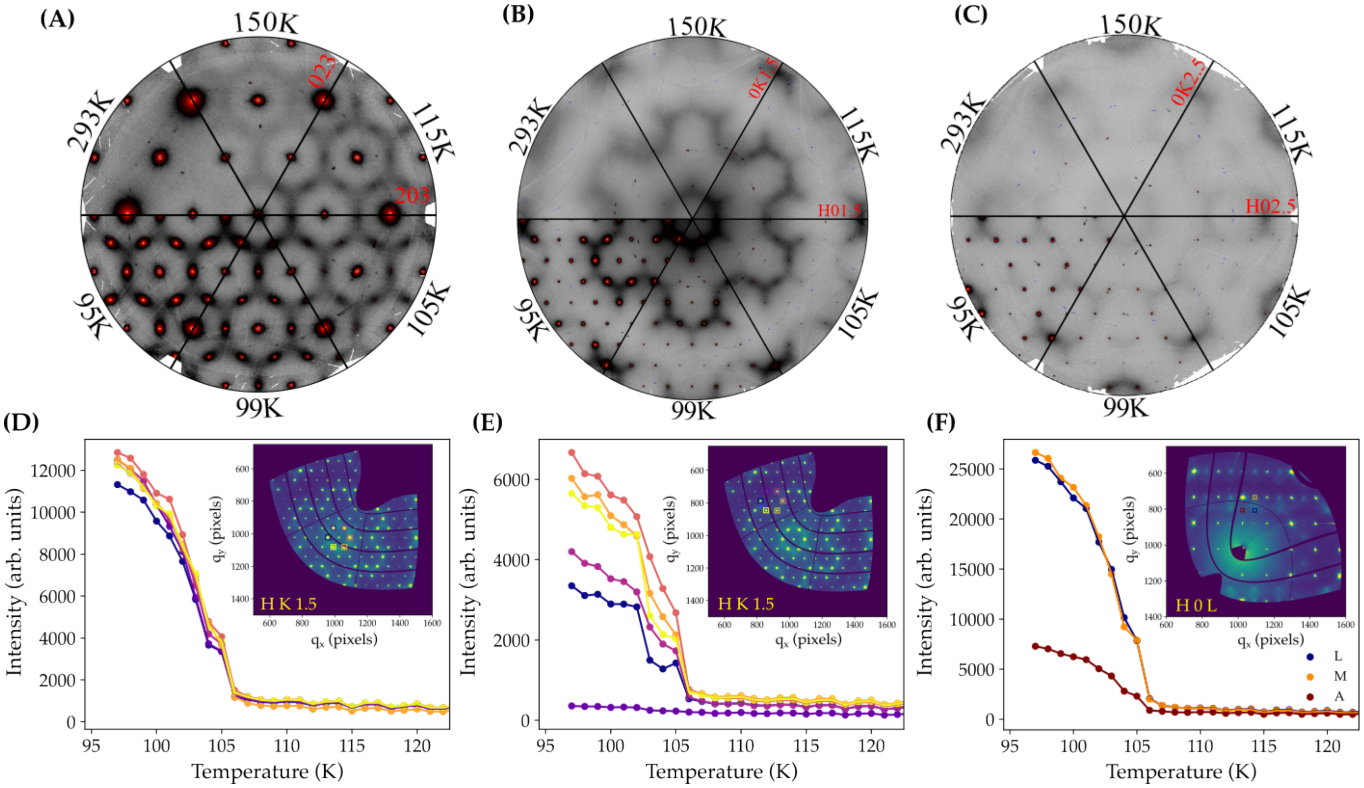

Supplementary Figure 17. DS maps of FeGe and integrated intensities of CDW peaks. (A) DS map of (h k 3). (B) DS map of (h k 1.5). (C) DS map of(h k 2.5). (D) Temperature evolution of the integrated intensities of the CDW peaks around the position (0 0 1.5). (E) Temperature evolution of the integrated intensities of the CDW peaks around the position (2 0 1.5). (F) Temperature evolution of the integrated intensities of the CDW peaks at A, M, and L in the h 0 l plane. In the inset of the panels (D-F), the squares are the integrated areas for each plot.

Supplementary Figs. 17 (D-F) display the integrated intensity of the CDW peaks and the corresponding diffuse precursors defined by the region of interest (ROI) in the insets. The intensities are strongly modulated in reciprocal space surrounding the Bragg positions.

B. Annealed FeGe

We have further studied the effect of annealing (72 hours) at higher temperatures; namely 330 ºC, 400 ºC and 550 ºC. The magnetization measurements identify the CDW transitions at around $\sim$ 100 K, see Supplementary Fig. 19, with the highest T_{CDW} for the 300 ºC annealed crystal (T_{CDW}=110 K), see Supplementary Fig. 21. On the other hand, the crystal annealed at 550 ºC shows a transition near room temperature and the magnetization curve resembles the reports in the literature [19]. This sample only develops DS down to 80 K, consistent with the absence of long-range charge order due to the creation of germanium vacancies throughout the FeGe kagome lattice, as reported by [20].

As we can see in Supplementary Fig. 20, the diffuse scattering, although with less signal-to-background ratio, is again localized along the M-L directions and also develops a hexagonal pattern at $T>T_{CDW}$ (annealed at 300 ºC). The spatial correlation of the CDW extends to $30.0 \pm 0.7\,\mathrm{nm}$ along M, $33.9 \pm 0.6\,\mathrm{nm}$ along L and $43.3 \pm 0.6\,\mathrm{nm}$ along A.

Supplementary Fig. 22 compares the (h k 2) DS of the annealed at 300 ºC and as-grown FeGe. Three features are visible: (1) the DS of the as-grown samples present a larger signal-to-noise ratio, (2) streaks of diffuse intensity appear in between Bragg peaks and cross the CDW DS at M and (3) the as-grown CDW peak width is more anisotropic than the annealed FeGe. We note

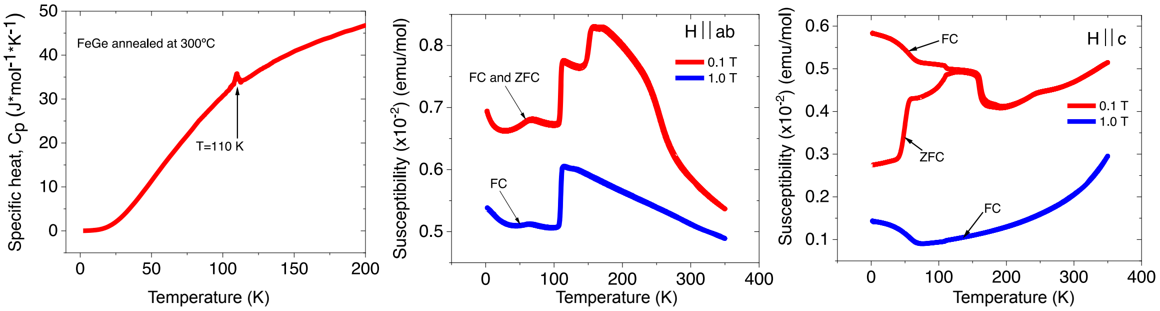

Supplementary Figure 18. Comparison of the specific heat, Cp, and the magnetization of FeGe annealed at 300 ºC, showing a sharp 1st order step anomaly from a transition at T_{CO} = 110 K.

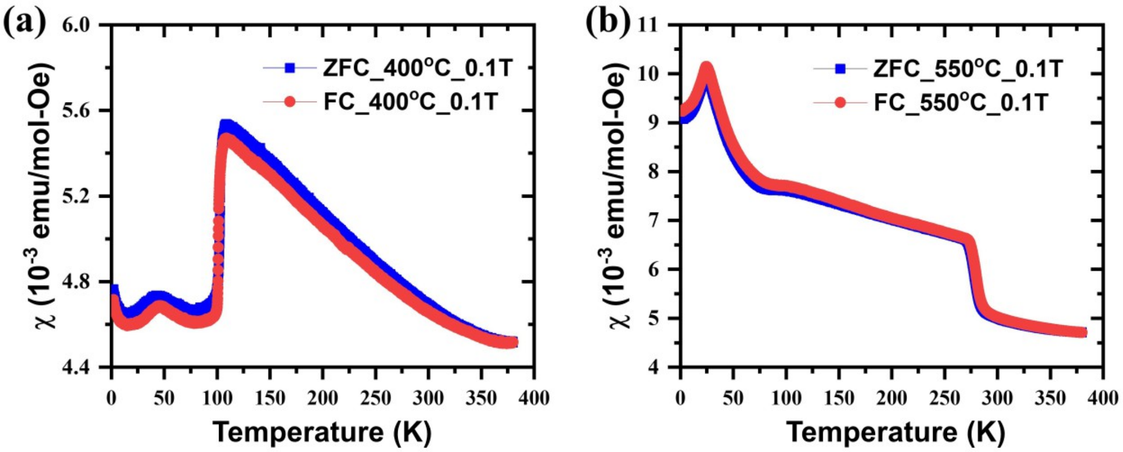

Supplementary Figure 19. Magnetic characterization of FeGe crystals annealed at 400 ºC (A) and 550 ºC (B) for 72 hours. The CDW transition is visible at around 100 K, but the 500 ºC annealed sample presents a magnetic transition at ∼300 K, presumably driven by the cubic FeGe.

that the lower intensity of the DS and the presence of orthorhombic domains preclude us from reaching a reliable comparison of the anisotropic width ratio between annealed FeGe and as-grown crystals.

Furthermore, at integer L and half-integer L-values, streaks of diffuse intensity, characteristic of orthorhombic domains, are visible between Bragg peaks. On the other hand, the hexagonal diffuse pattern is no longer visible at low temperatures, indicating that the annealing reduces the frustration between dimerized and undimerized phases at $T<T_{CDW}$.

The temperature dependence of the DS of FeGe annealed at 440 ºC and 550 ºC is also shown in Supplementary Fig. 20. We first note that the crystal with the highest T_{CDW} is the one annealed at 300 ºC, see Supplementary Fig. 21. Annealing at 400 ºC and 550 ºC gives a different result. The 400 ºC sample has a T_{CDW} of ∼100 K, similar to the as-grown crystal. The hexagonal rings are still present, although with less intensity, and the presence of orthorhombic domains is largely reduced. Comparing the 300 ºC and 400 ºC FeGe, it seems that the isotropy of the CDW peak correlates with the larger correlation length of the CDW peak. On the other hand, the FeGe sample annealed at 550 ºC presents very intense disorder-driven hexagonal rings, with a progressive increase of DS upon cooling and a suppression of the CDW. We do not observe CDW peaks in the 550 ºC annealed sample down to 80 K.

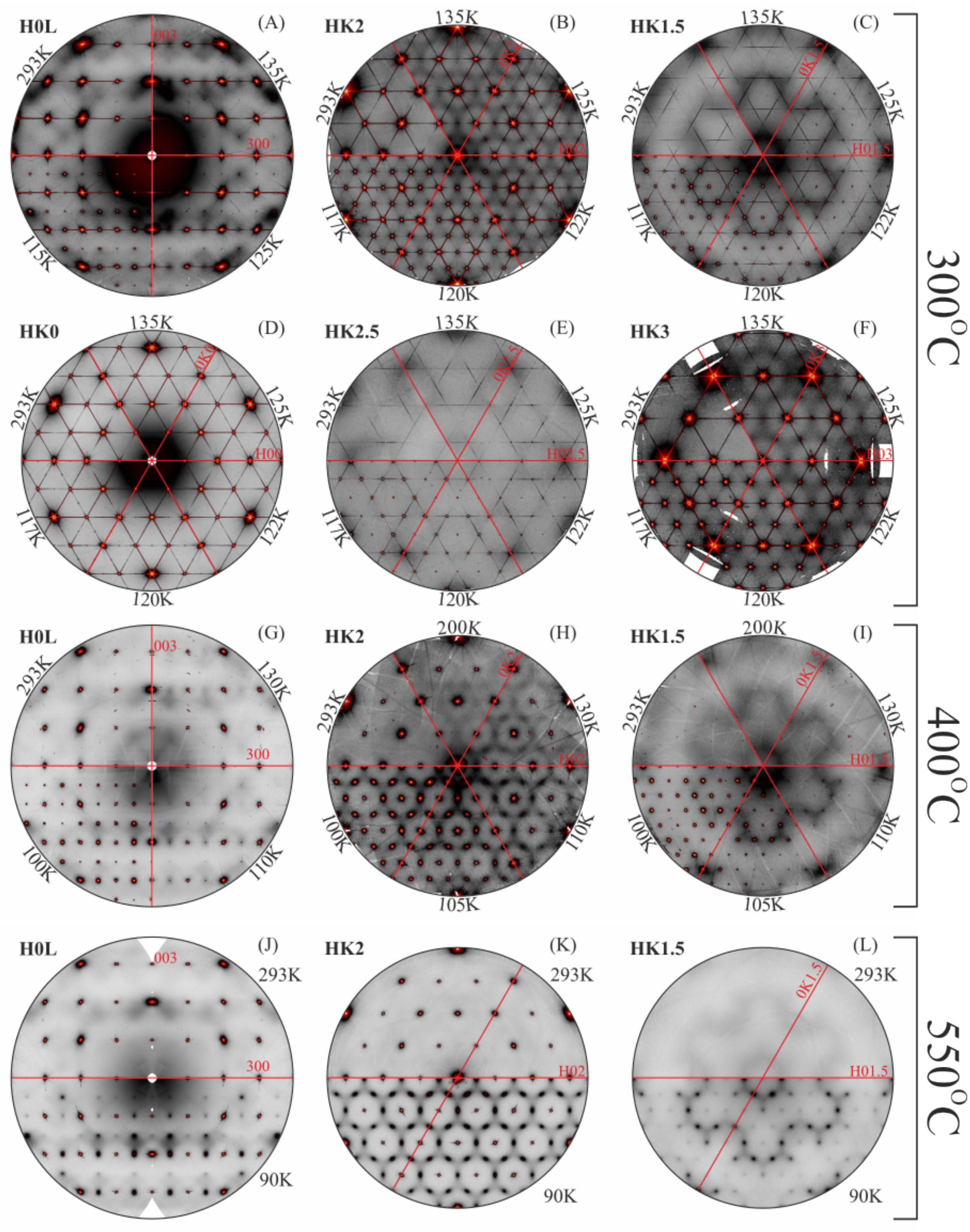

Supplementary Figure 20. DS maps of the annealed FeGe at different temperatures, 300 ºC, 400 ºC, and 550 ºC. The hexagonal diffuse rings are still present at $T>T_{\mathrm{CDW}}$ but absent for $T<T_{\mathrm{CDW}}$ for the sample treated at 300 ºC. Streaks of intensity are characteristic of orthorhombic domains. In addition, no anisotropic DS is observed at the M point. The DS maps of FeGe annealed at 400 ºC and 550 ºC develop more hexagonal diffuse intensity, in particular, the sample heated at 550 ºC, which shows indications for the presence of cubic FeGe in the magnetization data.

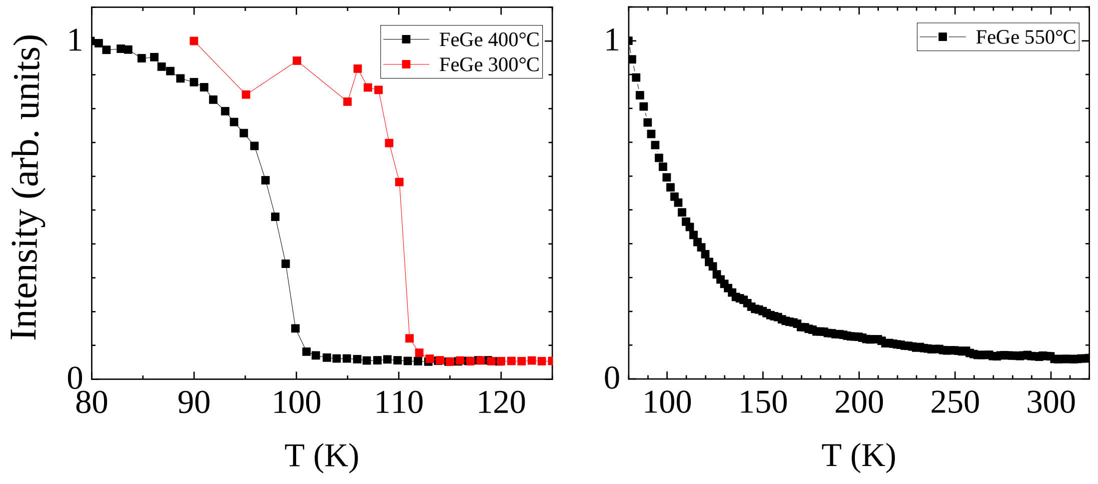

Supplementary Figure 21. Temperature dependence of the CDW for FeGe crystals annealed at 300 ºC, 400 ºC, and 550 ºC for 72 hours. The sample annealed at 300 ºC presents the highest T_{CDW}. On the other hand, FeGe annealed at 550 ºC, develops only DS down to 90 K.

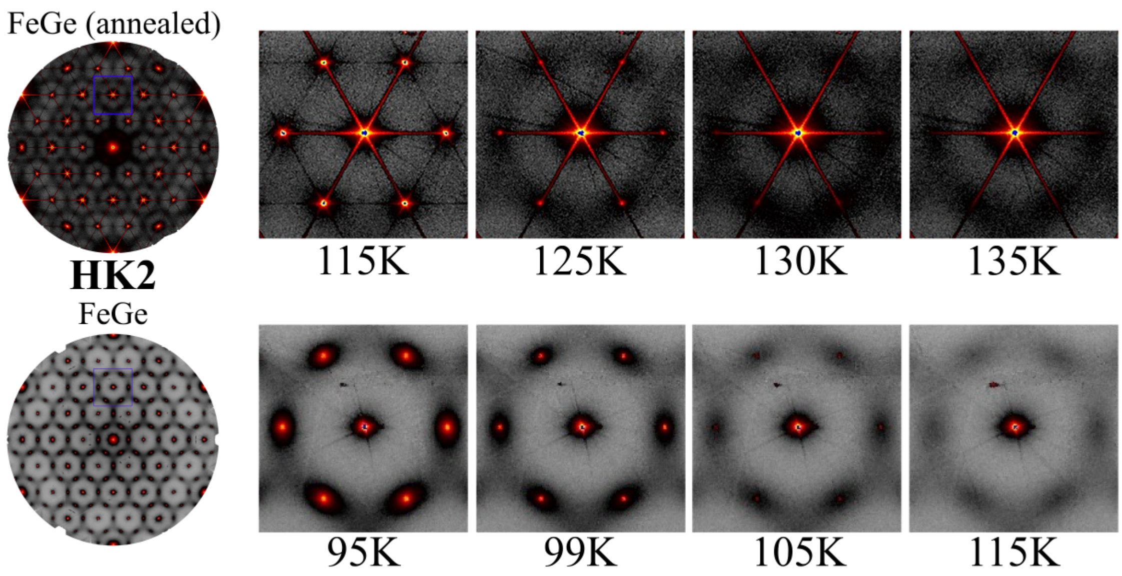

Supplementary Figure 22. Comparison of the (h k 2) plane of FeGe annealed at 300 ºC (A) and as-grown (B) FeGe crystals. (A) The DS of the annealed sample is at the background level and the diffuse signal isotropic around the CDW reflection.

C. $FeGe_{0.9}$

In this section, we present the results of Ge-deficient FeGe ($FeGe_{0.9}$). The Ge concentration was estimated from the energy dispersive analysis (EDX), Supplementary Fig. 23. The 10% Ge deficiency strongly modifies the magnetic behavior of the sample, closely resembling the magnetic curve of FeGe annealed at 550 ºC, but the AFM canting transition is still visible, Supplementary Fig. 24, and the compound develops order, although at lower temperature as compared with FeGe. Again, the transition around ∼200 K would be related to the formation of cubic FeGe.

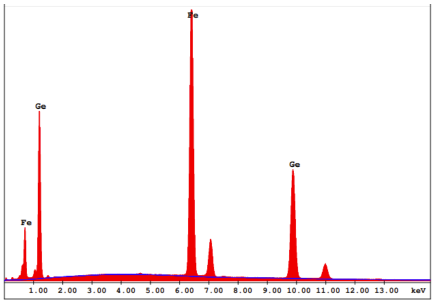

EDAX ZAF Quantification (Standardless)
Element Normalized
SEC Table : Default

Element	Wt %	At %	K-Ratio	Z	A	F
FeK	45.81	52.36	0.4803	1.0342	0.9690	1.0461
GeK	54.19	47.64	0.4956	0.9683	0.9445	1.0000
Total	100.00	100.00				

Element	Net Inte.	Bkgd Inte.	Inte. Error	P/B
FeK	11283.04	222.02	0.14	50.82
GeK	5323.02	137.08	0.20	38.83

Supplementary Figure 23. Energy dispersive analysis of Ge-deficient FeGe ($FeGe_{0.9}$).

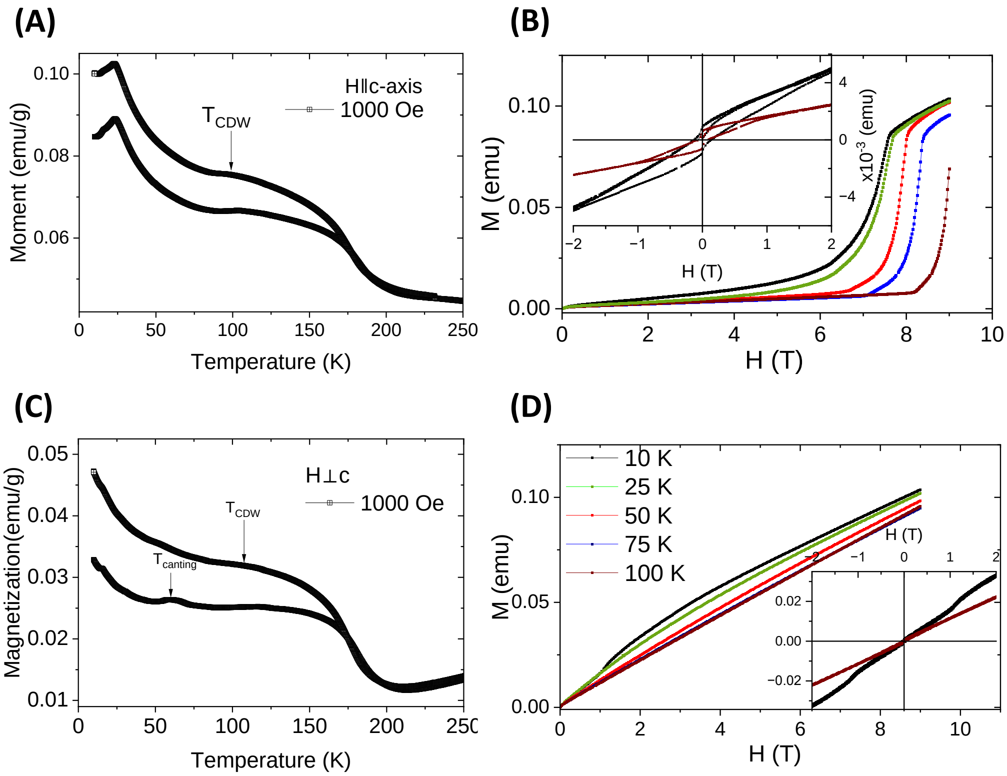

Supplementary Figure 24. Magnetic characterization of Ge-deficient FeGe (A) Temperature dependence of the magnetization for applied magnetic field (H) parallel to the *c*-axis (B) Magnetic field dependence of the magnetization for H parallel to *c*. Temperature (C) and magnetic field (D) dependence of the magnetization parallel to the *ab*-plane.

The DS maps of $FeGe_{0.9}$ show similar hexagonal diffuse patterns at integer and half-integer L-values as the stoichiometric FeGe. However, the intensity of the CDW peaks sharply drops below the T_{CDW}, see Supplementary Fig. 25, with an upturn

for some propagation vectors and even the complete disappearance of the CDW reflection at $q_{\mathbf{A}}$=(0 0 $\frac{3}{2}$). This behavior was not observed in $FeGe_{1.0}$. We have carried out energy-resolved inelastic x-ray scattering (XS) experiments to disentangle the elastic and inelastic contributions of the diffuse signal. As shown in Supplementary Fig. 26, the drop in intensity is mostly driven by the temperature dependence of the elastic central peak (CP) of the IXS spectrum.

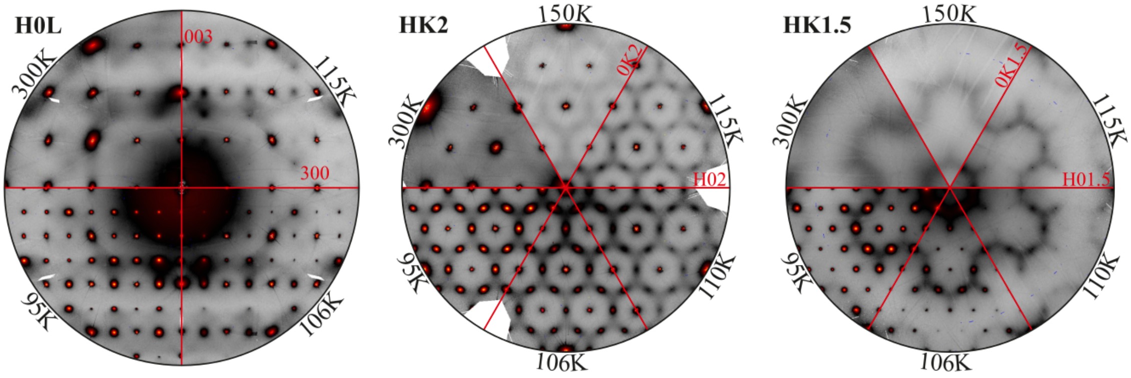

Supplementary Figure 25. Reciprocal space reconstructions of the Ge-deficient $FeGe_{0.9}$ diffuse maps.

The temperature dependence of the CP, and in particular the absence of the elastic signal at (0 0 $\frac{3}{2}$) at low temperature, is reminiscent of the reports charge ordered nickelates [21]. Although not explored in detail, we speculate on the role of quenched disorder or fluctuating charge order below T_{CDW}. Recently, Klemm et al. [20] reported a comprehensive characterization of FeGe annealed at 550 ºC, showing point defects (vacancies) on the trigonal Ge-sites throughout the sample suppress the formation of charge order. Such Ge vacancies might also be responsible for the anomalous drop of intensity below T_{CO} in $FeGe_{0.9}$.

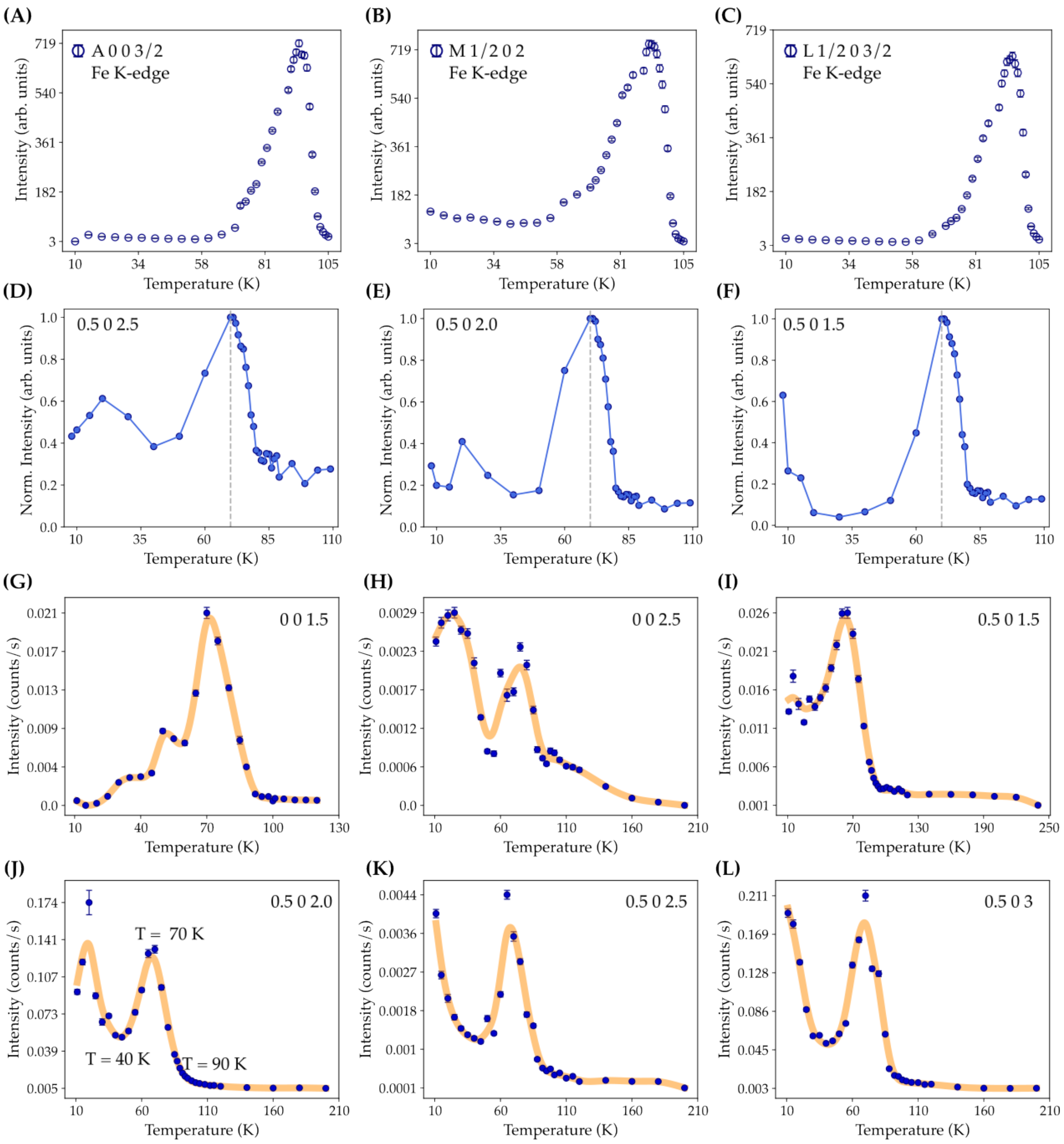

Supplementary Figure 26. (A-C) Temperature dependence of the CDW peaks A, M and L, respectively, measured at the Fe K-edge, 7.11 keV, in $FeGe_{0.9}$. (D-F) Temperature dependence of the DS integrated intensity of three different CDW peaks. (G-L) Temperature dependence of the integrated intensity of the elastic central peak (CP) of IXS for the Ge-deficient $FeGe_{0.9}$. The different transition temperatures are likely due to beam heating in resonant scattering as compared with IXS and diffuse scattering.

D. FeSn

We have searched for diffuse scattering in the antiferromagnetic FeSn with the in-plane spin polarization [22]. The absence of any type of diffuse pattern as a function of temperature demonstrates that the out-of-plane spin polarization of FeGe is responsible for the out-of-plane displacement of the trigonal Ge.

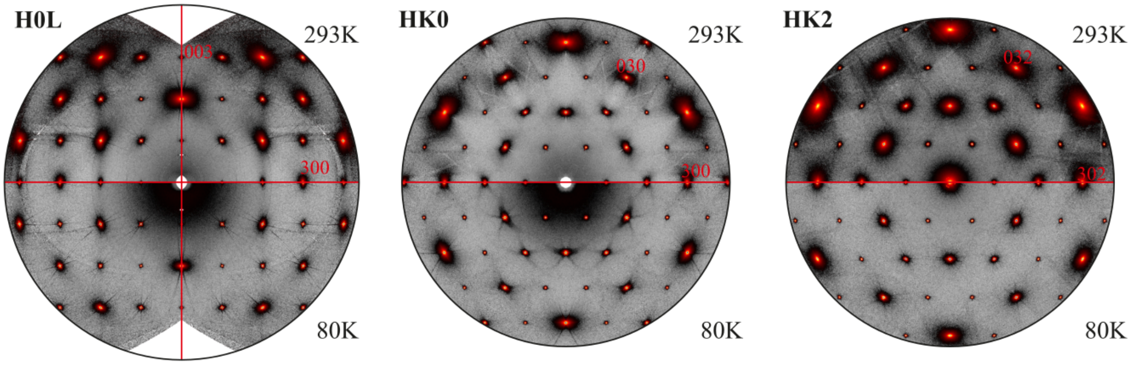

Supplementary Figure 27. Temperature dependence DS of FeSn. No diffuse signal is observed at either M, L, or A points.

The DS around the Bragg peaks at high temperatures result from the thermal excitations of phonon, which is. thermal diffuse scattering (TDS).

Supplementary Note 8. MONTE CARLO SIMULATION OF DS

In this section, we detail the Monte Carlo simulations of the DS, based on an effective Ising model that describes the dimerization of triangular Ge.

A. Ising model for Ge dimerization

The model (1) used in the main text is the Hamiltonian used in the Monte Carlo simulation for modeling the diffuse scattering. It is based on the Ising model of adjacent spins and is commonly used to describe binary substitution disorder with the 'spin' variables corresponding to one of two possible atoms occupying a lattice site. The simplest Ising model considers only the nearest neighbor interactions and an overall dipole. Empirically, we found that 3 nearest neighbor shells in the *ab*-plane and just the nearest neighbor shell along the *c*-direction were required to reproduce the diffuse scattering. The strength and direction of interacting spins are controlled through the c parameters. They represent an interaction strength or force constant between two spins along a particular direction; their size and sign therefore indicate the relative strength of interactions in different neighbor shells to drive the MC simulation towards a configuration that reproduces the diffuse scattering. In this work, the values were based on DFT energy calculations.

We first build an Ising model to describe the dimerization of triangular Ge and to achieve a certain microscopic realization via the Monte Carlo simulations. We use an Ising variable $\sigma_i = \pm 1$ to denote the dimerized ($\sigma_i = -1$) and undimerized ($\sigma_i = +1$) triangular Ge pair in the (non-CDW AFM phase) unit cell $\mathbf{R}_i$. By considering in-plane nearest neighbor (NN) coupling c_1, next NN (NNN) coupling c_2, 3rd-NN (3NN) coupling c_3, z-direction NN coupling c_4, and an effective magnetic field h, we build a model with the form

$$H = \sum_{\langle ij\rangle:NN} c_1\sigma_i\sigma_j + \sum_{\langle ij\rangle:NNN} c_2\sigma_i\sigma_j + \sum_{\langle ij\rangle:3NN} c_3\sigma_i\sigma_j + \sum_{\langle ij\rangle:z-NN} c_4\sigma_i\sigma_j + \sum_i h\sigma_i + E_0. \tag{5}$$

where E_0 is a constant and $\langle ij\rangle$ means each ij pair counts only once. The magnetic field term h is added because the fully dimerized and undimerized configurations have different energies.

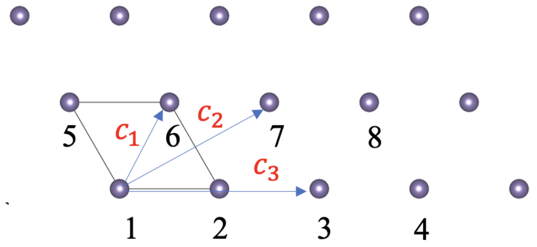

Supplementary Figure 28. The illustration of the coupling parameters used in the effective Ising model in Eq. (5). In the figure, $c_{1,2,3}$ are in-plane NN, NNN, and 3NN coupling parameters. The numbers 1-8 label the eight Ge pairs in a 4×2 in-plane supercell of the non-CDW unit cell. The black lines mark the non-CDW unit cell.

We then fit the parameters from DFT. We choose a $4\times2\times2$ supercell of the non-CDW AFM unit cell, which contains $4\times2\times2$ Ge pairs. As shown in table III, we consider 7 in equivalent dimer configurations and compute their averaged magnetic moments and total energies in DFT. The parameters in the Ising model Eq. (5) are fitted using the DFT data, with their values summarized in table IV.

B. Monte Carlo simulations

With the effective Ising model derived based on DFT, we perform Monte Carlo simulations to obtain simulated diffuse scattering patterns. In the Monte Carlo simulation, moves are accepted if a random number (0-1) is less than $exp(-\Delta E/k_B T)$ with $T = 80K$. The low-temperature unit cell (10 × 10 × 8 Å) was expanded to a 16 × 16 × 16 supercell. ~5% dimers were added randomly and the MC simulations were run until convergence.

The real space configuration of the minimum energy supercell is depicted in Fig. 29(A) where the red and gray balls stand for dimerized and non-dimerized phases. Most of the atoms are highly ordered (circled in black) but there are some defect areas where the ordering is not complete (circled in blue). These configurations converged to 37.5% non-dimers and 62.5% dimers.

Dimer configuration	$\bar{\mu}_{Fe}/\mu_B$	DFT total energy (meV)	Fitted energy (meV)
$-+-+,++++,\quad -+-+,++++$	1.44	0.0	0.0
$++++,++++,\quad ++++,++++$	1.39	835.4249	772.5727
$-+++,-+++,\quad -+++,-+++$	1.44	997.3268	997.3268
$-+++,++-+,\quad -+++,++-+$	1.43	667.4307	667.4307
$-+++,++++,\quad -+++,++++$	1.41	517.8678	517.8678
$-+-+,++++,\quad ++++,++++$	1.41	614.8985	740.6027
$-+-+,++++,\quad ++++,+-+-$	1.44	771.4848	708.6327

Supplementary Table III. The computed total energies from DFT for different dimer configurations. For each dimer configuration, $+$ $(-)$ denotes the dimerized (undimerized) triangular Ge pair. The first 8 $\pm$ denotes the 8 Ge pairs marked in Fig. 28 on the first layer in the $4 \times 2 \times 2$ supercell, and the second 8 $\pm$ denotes the second layer. The second column of $\bar{\mu}_{Fe}$ is the averaged magnitude of magnetic moment on Fe atoms. The third column is the computed DFT total energy, while the last column is the fitted energy using Eq. (5). Remark that the configurations with more dimerized Ge have larger magnetic moments and much higher total energies, and are not used in the fitting.

c_1	c_2	c_3	c_4	h	E_0
45.885	25.267	-8.224	-44.290	-330.340	3746.100

Supplementary Table IV. The fitted value of parameters in the Ising model Eq. (5) based on the *ab initio* data in table III. All numbers are given in meV.

The diffuse scattering calculated from these configurations using DFT parameters (Bragg peaks have been removed) is plotted in Figs. 29 (B) and 30 (A). The diffuse scattering is calculated using the program Scatty by Fourier transforming all the atomic coordinates of the atomic positions. In Figs. 30 (A-F), the highly intense Bragg nodes at the A point are removed.

The DFT values reproduce the DS at the M and L points, Figs. 30 (A-B). Moreover, upon manually tuning the c_1 and c_2 values, we can also simulate the shape of the anisotropic DS at the M point at 80 K and 100 K. This shows that the c_i's parameters that describe the nearest neighbor interaction between dimerized and undimerized trigonal Ge are temperature dependent.

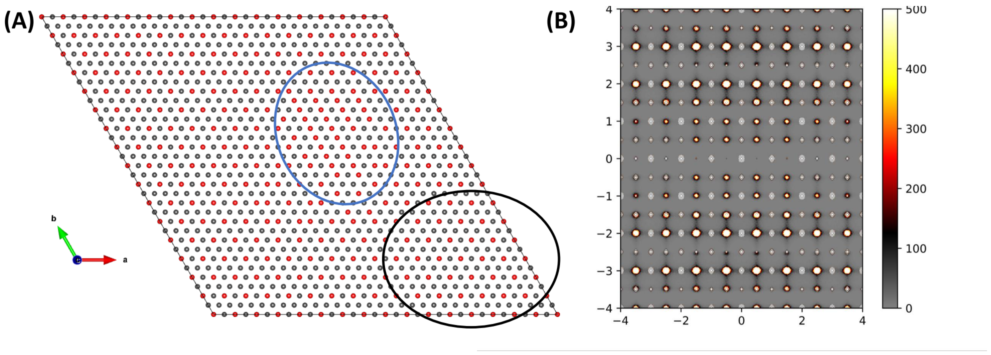

Supplementary Figure 29. (A) Converged MC simulation of a supercell of FeGe using the Ising model defined in 5. The circles highlight the 2 typical atomic arrangements. (B) (h 0 l) calculated DS map of FeGe showing the diffuse signal at the A, M, and L points. Bragg peaks are omitted.

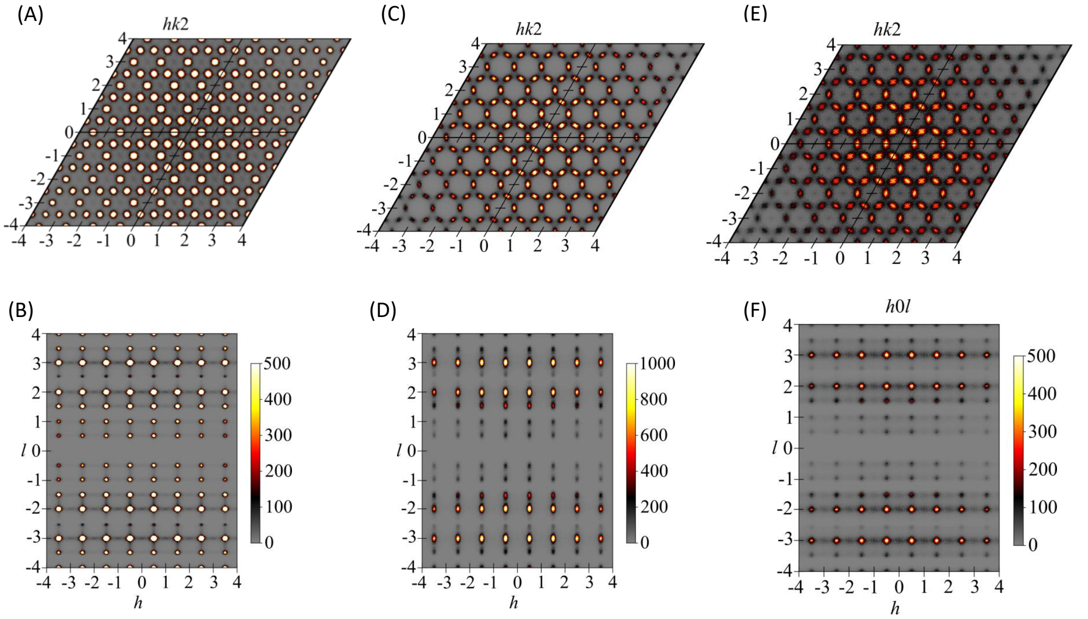

Supplementary Figure 30. Simulated h k 2 and h 0 l DS maps. (A-B) h k 2 and h 0 l DS obtained by minimizing the Ising Hamiltonian using the c_i values obtained from DFT, c_1= 45.885 meV, c_2= 25.267 meV, c_3= -8.224, meV c_4= -44.290 meV, h = -330.340 meV (T=80 K). (C-D) h k 2 and h 0 l DS for c_1= 165.886 meV, c_2= 25.267 meV, c_3= -8.224, meV c_4= -44.290 meV (T=80 K). (E-F) h k 2 and h 0 l DS c_1= 165.886 meV, c_2= 0 meV, c_3= -8.224, meV c_4= -44.290 meV (T=100 K). The simulations do not include the in-plane atomic displacements, which are responsible for the absence of DS at l=integer+$\frac{1}{2}$. See main text.

Supplementary Note 9. BOND ORDER (BO) CORRELATION ANALYSIS

In this section, we describe the analysis of the bond-order correlation analysis. For a discrete set of particles in the real space, one can define a local ordering between nearest neighbors as

$$\Psi_6(\mathbf{r}_k) = \frac{1}{N_k}\sum_{j=1}^{N_k} \mathrm{e}^{i6\theta_{kj}}, \tag{6}$$

where N_k is the number of nearest neighbors of the $k-$particle at position $\mathbf{r}_k$ and θ_{kj} defines the angle the k-j bond (Fig. 4(I) in the main text). For a particle at $\boldsymbol{r}_k$, the six-bond order correlation function at a distance $|\boldsymbol{r}-\boldsymbol{r_k}|$ is given by

$$G_6(|\boldsymbol{r}-\boldsymbol{r_k}|) = \frac{1}{N_{|\boldsymbol{r}-\boldsymbol{r_k}|}}\sum_{j}^{N_{|\boldsymbol{r}-\boldsymbol{r_k}|}} \Psi_6(\boldsymbol{r}_k)\Psi_6^*(\boldsymbol{r}_j), \tag{7}$$

where the sum goes over all the particles at a distance $|\boldsymbol{r}-\boldsymbol{r_k}|$ with respect to $\boldsymbol{r_k}$. Summing over all the particles in the system, we can define a total $G_6(\boldsymbol{r})$,

$$G_6(\boldsymbol{r}) = \frac{1}{N_r}\sum_{<j,k>}^{N_r} \Psi_6(\boldsymbol{r}_k)\Psi_6^*(\boldsymbol{r}_j), \tag{8}$$

- where N_r goes over any pair of particles which are at a distance $\boldsymbol{r}$. To compute the $G_6(\boldsymbol{r})$ correlation function, first, we need to get the real-space charge distribution from the diffuse scattering maps. The intensity I of a diffuse scattering map is proportional to the square of the structure factor,

$$I \propto |S\,(q)\,|^2, \tag{9}$$

- where $\boldsymbol{q}$ is a vector in the reciprocal space. The real-space charge distribution is defined as the real part of the Fourier transform of the structure factor,

$$\rho\,(\boldsymbol{r}) = \frac{1}{V_{cell}}\sum_{\boldsymbol{q}}^{N} |S\,(\boldsymbol{q})\,|\cos\left(2\pi\,(\boldsymbol{q}\cdot\boldsymbol{r}) + \Phi\,(\boldsymbol{q})\right), \tag{10}$$

- where $\boldsymbol{r}$ is the position vector in the real space and $\Phi\,(\boldsymbol{q})$ is a random phase $S\,(\boldsymbol{q})$ for a given $\boldsymbol{q}$ [23–25].

As Supplementary Eq.8 is defined over a discrete set of particles, we make a discretization of the continuous real-space charge distributions by defining the local maxima as particles. Then, we introduce a Voronoi tessellation in the discrete set of particles to define the concept of neighbor. Given a set of points (particle's positions) $\{p_1, p_2, ..., p_N\}$, each point p_i has a Voronoi cell associated. This cell consists of any point in the Euclidean space for which p_i is the nearest site of the set of points. All the Voronoi cells together form the Voronoi tessellation and any pair of cells which share a boundary will correspond to a pair of points which are neighbors. The geometrical construction of this diagram is equivalent to the one used to get a Wigner-Seitz cell. Once we have introduced the concept of neighbor in the discrete set of particles, we can compute Eqs. 6,7,8.

Supplementary Fig. 31 summarizes the real space charge density considering different types of CDW peak shape/anisotropy for a 6-fold symmetry. In Figs. 31 (A, C, E), where the CDW peaks are rather sharp, we can discretize the charge density to perform a Voronoi analysis, however, at high temperature as is the case of Supplementary Fig. 31 (I), the transformation from a continuous field to a discrete set of particles, where there is no well-defined six-fold symmetry, the charge density is well capture by means of a discretization analysis. Supplementary Figs. 32 (A, D, G, J) corresponds to the experimental maps of diffuse scattering we have used to perform the G_6 analysis in Fig. 4 of the main text. For each one of these DS, we have performed the Fourier transform to real space (see Figs. (B, E, H, K)) via Supplementary Eq. 10. After the discretization, we got the Voronoi tessellation and made the bond-order correlation function via Supplementary Eq. 8.

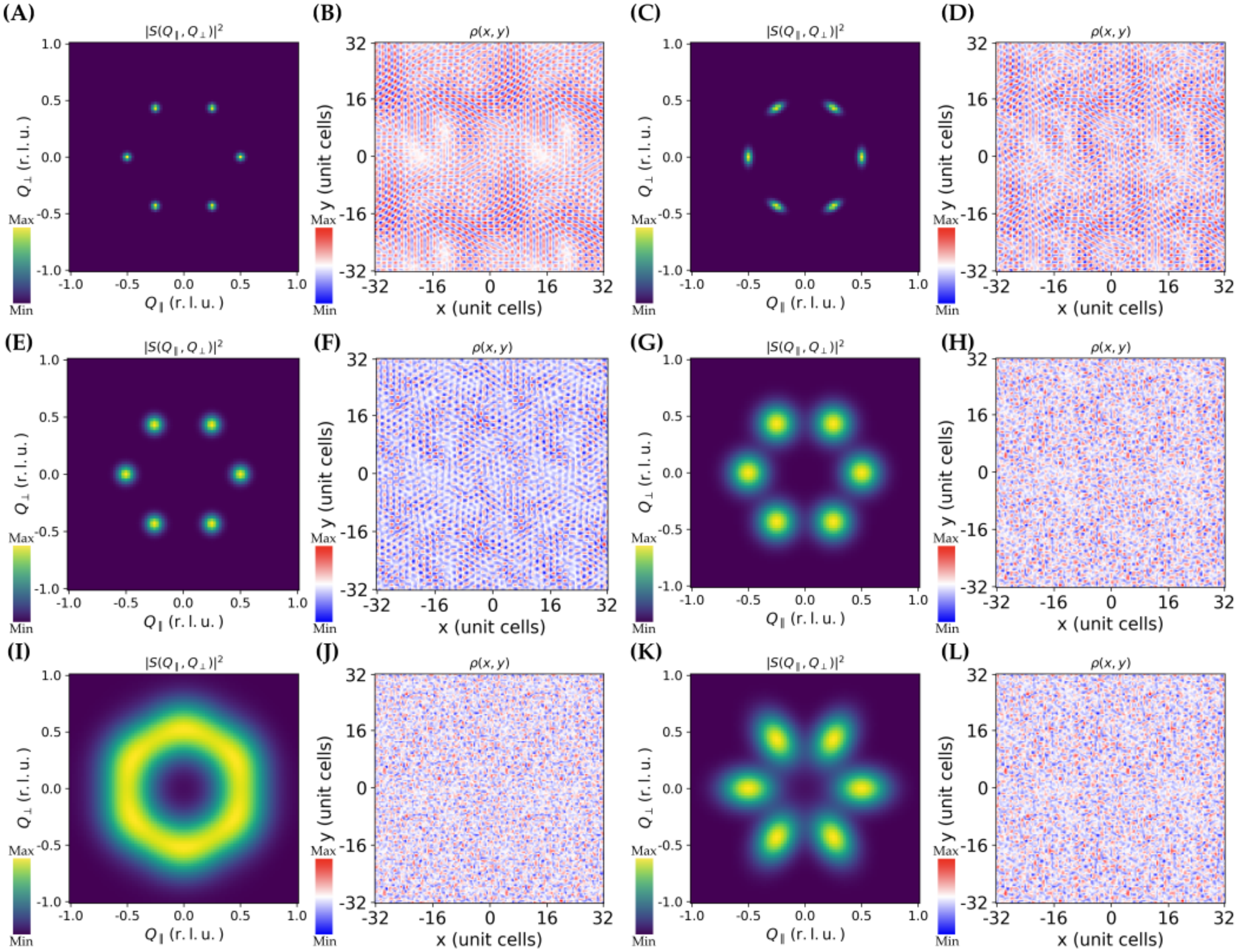

Supplementary Figure 31. (A) Simulated DS map by using a Gaussian Line shape centered at each CDW peak and line widths $[\sigma_x,\ \sigma_y]$ equal to $[0.002,\ 0.002]$ r.l.u.. (B) Fourier transform of (A). Each pair from (C-L) are the same varying the line widths of the Gaussians. (C-D) DS and its Fourier transform where the Gaussians have a line width of $[0.01,\ 0.002]$ r.l.u.. (E-F) DS and its Fourier transform where the Gaussians have a line width of $[0.01,\ 0.01]$ r.l.u.. (G-H) DS and its Fourier transform where the Gaussians have a line width of $[0.05,\ 0.05]$ r.l.u.. (I-J) DS and its Fourier transform where the Gaussians have a line width of $[0.3,\ 0.3]$ r.l.u.. (K-L) DS and its Fourier transform where the Gaussians have a line width of $[0.05,\ 0.1]$ r.l.u.. For each Fourier transform it was used the same random phase matrix.

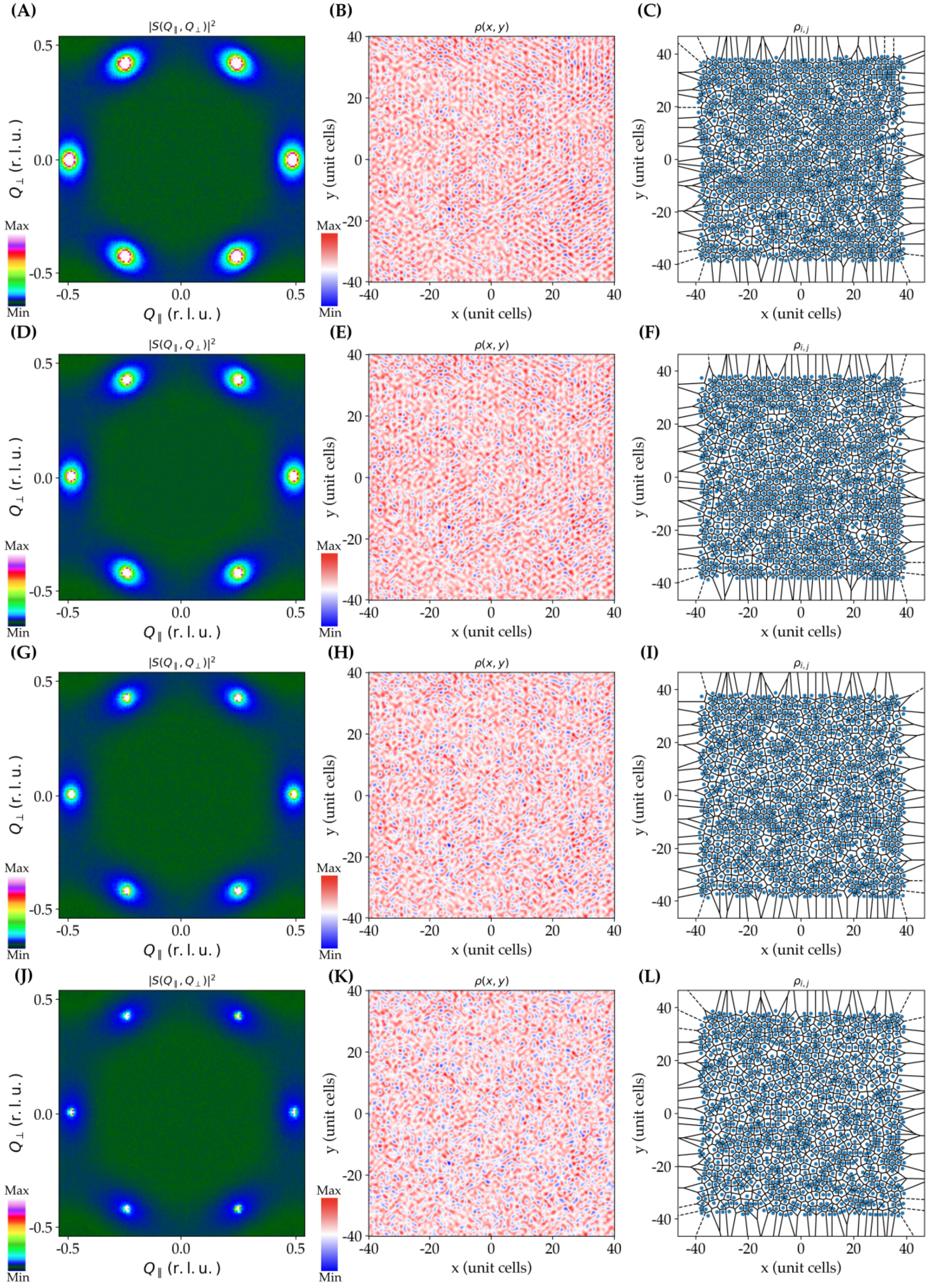

Supplementary Figure 32. (A, D, G, J) Diffuse Scattering maps of the plane (H K 2.0) around the point (0 0 2.0) for $T = 95,\ 97,\ 99,\ 101\,\mathrm{K}$, respectively. The Bragg point just in the point (0 0 2.0) has been screened by introducing random Gaussian noise with an average and standard deviation equal to the thermal background. (B, E, H, K) Real-space charge distribution for the diffuse scattering maps of (A, D, G, J), respectively. (C, F, I, L) Voronoi tessellation of the local maxima of the charge distribution in (B, E, H, K), respectively.

[1] Xiaokun Teng, Lebing Chen, Feng Ye, Elliott Rosenberg, Zhaoyu Liu, Jia-Xin Yin, Yu-Xiao Jiang, Ji Seop Oh, M. Zahid Hasan, Kelly J. Neubauer, Bin Gao, Yaofeng Xie, Makoto Hashimoto, Donghui Lu, Chris Jozwiak, Aaron Bostwick, Eli Rotenberg, Robert J. Birgeneau, Jiun-Haw Chu, Ming Yi, and Pengcheng Dai, "Discovery of charge density wave in a kagome lattice antiferromagnet," Nature **609**, 490–495 (2022).

[2] Xiaokun Teng, Ji Seop Oh, Hengxin Tan, Lebing Chen, Jianwei Huang, Bin Gao, Jia-Xin Yin, Jiun-Haw Chu, Makoto Hashimoto, Donghui Lu, Chris Jozwiak, Aaron Bostwick, Eli Rotenberg, Garrett E. Granroth, Binghai Yan, Robert J. Birgeneau, Pengcheng Dai, and Ming Yi, "Magnetism and charge density wave order in kagome FeGe," Nature Physics **19**, 814–822 (2023).

[3] Georg Kresse and Jürgen Furthmüller, "Efficiency of ab-initio total energy calculations for metals and semiconductors using a plane-wave basis set," Computational materials science **6**, 15–50 (1996).

[4] Georg Kresse and JJPRB Hafner, "Ab initio molecular dynamics for open-shell transition metals," Physical Review B **48**, 13115 (1993).

[5] Georg Kresse and Jürgen Hafner, "Ab initio molecular dynamics for liquid metals," Physical review B **47**, 558 (1993).

[6] Georg Kresse and Jürgen Hafner, "Ab initio molecular-dynamics simulation of the liquid-metal–amorphous-semiconductor transition in germanium," Physical Review B **49**, 14251 (1994).

[7] Georg Kresse and Jürgen Furthmüller, "Efficient iterative schemes for ab initio total-energy calculations using a plane-wave basis set," Physical review B **54**, 11169 (1996).

[8] John P Perdew, Kieron Burke, and Matthias Ernzerhof, "Generalized gradient approximation made simple," Physical review letters **77**, 3865 (1996).

[9] Nicola Marzari and David Vanderbilt, "Maximally localized generalized wannier functions for composite energy bands," Physical review B **56**, 12847 (1997).

[10] Ivo Souza, Nicola Marzari, and David Vanderbilt, "Maximally localized wannier functions for entangled energy bands," Physical Review B **65**, 035109 (2001).

[11] Nicola Marzari, Arash A Mostofi, Jonathan R Yates, Ivo Souza, and David Vanderbilt, "Maximally localized wannier functions: Theory and applications," Reviews of Modern Physics **84**, 1419 (2012).

[12] Giovanni Pizzi, Valerio Vitale, Ryotaro Arita, Stefan Blügel, Frank Freimuth, Guillaume Géranton, Marco Gibertini, Dominik Gresch, Charles Johnson, Takashi Koretsune, et al., "Wannier90 as a community code: new features and applications," Journal of Physics: Condensed Matter **32**, 165902 (2020).

[13] Yi Jiang, Haoyu Hu, Dumitru Călugăru, Claudia Felser, Santiago Blanco-Canosa, Hongming Weng, Yuanfeng Xu, and B Andrei Bernevig, "Kagome materials ii: Sg 191: FeGe as a lego building block for the entire 1: 6: 6 series: hidden d-orbital decoupling of flat band sectors, effective models and interaction hamiltonians," arXiv preprint arXiv:2311.09290 (2023).

[14] QuanSheng Wu, ShengNan Zhang, Hai-Feng Song, Matthias Troyer, and Alexey A Soluyanov, "Wanniertools: An open-source software package for novel topological materials," Computer Physics Communications **224**, 405–416 (2018).

[15] Q Zheng, "Vasp band unfolding," URL https://github. com/QijingZheng/VaspBandUnfolding (2018).

[16] Voicu Popescu and Alex Zunger, "Extracting e versus k effective band structure from supercell calculations on alloys and impurities," Physical Review B **85**, 085201 (2012).

[17] Mitsuaki Kawamura, "Fermisurfer: Fermi-surface viewer providing multiple representation schemes," Computer Physics Communications **239**, 197–203 (2019).

[18] T.R. Welberry, Diffuse X-Ray Scattering and Models of Disorder, IUCr monographs on crystallography (Oxford University Press, 2022).

[19] Xueliang Wu, Xinrun Mi, Long Zhang, Chin-Wei Wang, Nour Maraytta, Xiaoyuan Zhou, Mingquan He, Michael Merz, Yisheng Chai, and Aifeng Wang, "Annealing-tunable charge density wave in the magnetic kagome material fege," Phys. Rev. Lett. **132**, 256501 (2024).

[20] Mason L. Klemm, Saif Siddique, Yuan-Chun Chang, Sijie Xu, Yaofeng Xie, Tanner Legvold, Mehrdad T. Kiani, Feng Ye, Huibo Cao, Yiqing Hao, Wei Tian, Hubertus Luetkens, Masaaki Matsuda, Douglas Natelson, Zurab Guguchia, Chien-Lung Huang, Ming Yi, Judy J. Cha, and Pengcheng Dai, "Vacancy-induced suppression of cdw order and its impact on magnetic order in kagome antiferromagnet fege," (2024), arXiv:2410.13994 [cond-mat.str-el].

[21] Alessandro Ricci, Nicola Poccia, Gaetano Campi, Shrawan Mishra, Leonard Müller, Boby Joseph, Bo Shi, Alexey Zozulya, Marcel Buchholz, Christoph Trabant, James C. T. Lee, Jens Viefhaus, Jeroen B. Goedkoop, Agustinus Agung Nugroho, Markus Braden, Sujoy Roy, Michael Sprung, and Christian Schüßler-Langeheine, "Measurement of spin dynamics in a layered nickelate using x-ray photon correlation spectroscopy: Evidence for intrinsic destabilization of incommensurate stripes at low temperatures," Phys. Rev. Lett. **127**, 057001 (2021).

[22] Mingu Kang, Jonathan Pelliciari, Alex Frano, Nicholas Breznay, Enrico Schierle, Eugen Weschke, Ronny Sutarto, Feizhou He, Padraic Shafer, Elke Arenholz, Mo Chen, Keto Zhang, Alejandro Ruiz, Zeyu Hao, Sylvia Lewin, James Analytis, Yoshiharu Krockenberger, Hideki Yamamoto, Tanmoy Das, and Riccardo Comin, "Evolution of charge order topology across a magnetic phase transition in cuprate superconductors," Nature Physics **15**, 335–340 (2019).

[23] R. Comin, R. Sutarto, E. H. da Silva Neto, L. Chauviere, R. Liang, W. N. Hardy, D. A. Bonn, F. He, G. A. Sawatzky, and A. Damascelli, "Broken translational and rotational symmetry via charge stripe order in underdoped $YBa_2Cu_3O_{6+y}$," Science **347**, 1335–1339 (2015).

[24] B. V. Fine, "Comment on 'broken translational and rotational symmetry via charge stripe order in underdoped $YBa_2Cu_3O_{6+y}$'," Science **351**, 235–235 (2016).

[25] R. Comin, R. Sutarto, E. H. da Silva Neto, L. Chauviere, R. Liang, W. N. Hardy, D. A. Bonn, F. He, G. A. Sawatzky, and A. Damascelli, "Response to comment on 'broken translational and rotational symmetry via charge stripe order in underdoped $YBa_2Cu_3O_{6+y}$'," Science **351**, 235–235 (2016).
